# PIPKIγ promotes non-homologous end joining through LIG4 to enhance radiotherapy resistance in triple-negative breast cancer

**DOI:** 10.1038/s41419-025-07894-5

**Published:** 2025-07-31

**Authors:** Wenge Dong, Haiping Zhang, Zhigang Zhuang, Ying Jiang

**Affiliations:** 1 Department of Breast Surgery, Shanghai Key Laboratory of Maternal Fetal Medicine, Shanghai Institute of Maternal-Fetal Medicine and Gynecologic Oncology, Shanghai First Maternity and Infant Hospital, School of Medicine, Tongji University, Shanghai, China; 2 Shanghai Key Laboratory of Maternal Fetal Medicine, Clinical and Translational Research Center of Shanghai First Maternity and Infant Hospital, Frontier Science Center for Stem Cell Research, School of Life Sciences and Technology, Tongji University, Shanghai, China

**Keywords:** Breast cancer, Breast cancer

## Abstract

Triple-negative breast cancer (TNBC) is an aggressive subtype of breast cancer with poor prognosis and limited therapeutic options. DNA damage-based radiotherapy plays a significant role in the treatment of TNBC. However, radioresistance continues to pose a significant challenge, often resulting in the unsuccessful outcome of radiation therapy for patients with TNBC. Type I gamma phosphatidylinositol phosphate kinase (PIPKIγ), a key enzyme in phosphoinositide metabolism, is associated with poor prognostic outcomes in TNBC. Here, we discovered that PIPKIγ overexpression significantly boosts nonhomologous end joining (NHEJ), a principal mechanism for repairing DNA double-strand breaks (DSBs). This enhancement of NHEJ confers increased radioresistance in TNBC. At the molecular level, PIPKIγ directly interacts with LIG4, a crucial NHEJ component, and strengthens its interaction with XRCC4, a key regulator of LIG4 nuclear translocation. This facilitates LIG4’s nuclear translocation, improving DNA repair efficiency and genomic stability in TNBC cells. Furthermore, elevated PIPKIγ levels enhance radioresistance in TNBC cells and tumors in xenograft models, whereas depleting PIPKIγ has the opposite effects. These insights reveal a new mechanism by which PIPKIγ promotes radioresistance through the facilitation of DSBs repair in TNBC. Collectively, our findings suggest that the PIPKIγ-LIG4 interaction represents a potential therapeutic target for improving radiotherapy efficacy in TNBC patients.

## Introduction

Triple-negative breast cancer (TNBC) is an aggressive type of breast cancer (BC), defined by the absence of estrogen receptor (ER), progesterone receptor (PR), and human epidermal growth factor receptor 2 (HER2) [1]. This subtype is characterized by a high potential for metastasis, increased recurrence rates, and a generally poor prognosis [1–4]. Without these receptors, TNBC cannot be treated with the targeted therapies that are successful for other breast cancer types, such as those with positive ER or HER2 status, thus considerably reducing the therapeutic alternatives [5]. Radiotherapy (RT) is a crucial part of the limited treatments for BC, particularly in TNBC patients, playing a key role in local control [6]. Compared to patients who did not receive postoperative RT, those who received adjuvant RT exhibited a significant reduction in the 10-year recurrence rate and a notable improvement in the 15-year survival rate [7].

RT is a widely used cancer treatment that exerts its therapeutic effect primarily by inflicting lethal DNA damage on tumor cells. DNA double-strand breaks (DSBs) are considered the most deleterious type of DNA damage and a primary cause of cell death during radiotherapy [8]. However, some cancers exhibit significant radioresistance due to their efficient and robust DNA repair mechanisms [9–13]. Therefore, therapies targeting the DNA DSBs repair machinery have the potential to improve the efficacy of RT [14]. TNBC is more prone to developing radioresistance than other BC subtypes [6, 15]. Elucidating the molecular mechanisms driving therapeutic resistance is crucial for improving TNBC treatment outcomes.

The two principal pathways for repairing DSBs are non-homologous end joining (NHEJ) and homologous recombination (HR), both of which are vital for cell survival [16]. NHEJ, which operates throughout the cell cycle, involves the direct ligation of DNA ends and is facilitated by key proteins such as the Ku70/80 heterodimer, DNA-PKcs, XRCC4, and LIG4 [17]. Among these NHEJ factors, LIG4 is indispensable for the final ligation step following DSBs, a process critical for completing NHEJ repair and maintaining genomic integrity. LIG4 deficiency results in severe clinical conditions like LIG4 syndrome, which is characterized by immunodeficiency, growth defects, and radiosensitivity due to impaired DNA repair [18]. NHEJ has been suggested to confer radioresistance through different regulatory mechanisms. In esophageal squamous cell carcinoma, VAV2 expedites the assembly of Ku70/Ku80 heterodimers at DNA damage sites, bolstering NHEJ repair of ionizing radiation-induced DNA lesions and contributing to radioresistance [19]. Similarly, in nasopharyngeal carcinoma, PPP1CC engages with Ku70/Ku80 heterodimers and activates DNA-PKcs, enhancing NHEJ-mediated DNA repair and fostering radioresistance through the promotion of DNA-PK holoenzyme formation [20]. In TNBC, BUB1 enhances radioresistance by recruiting and activating phosphorylated DNA-PKcs at DNA DSBs, thereby improving tumor survival post-radiotherapy [9]. Moreover, METTL1-mediated m7G tRNA modification specifically regulates the translation of LIG4 and other DNA-dependent protein kinase subunits, which have an increased frequency of m7G-related codons following ionizing radiation exposure, ultimately amplifying radiotherapy resistance in hepatocellular carcinoma [21]. In lung cancer, PKP2 stabilizes β-catenin to induce LIG4 transcription, thereby enhancing NHEJ repair and promoting radioresistance [22]. While the role of LIG4 in NHEJ repair and radiotherapy resistance is well-documented across various types of cancers, its specific influence on NHEJ in TNBC warrants further exploration.

Type I gamma phosphatidylinositol phosphate kinase (PIPKIγ) is a key enzyme in phosphoinositide metabolism, catalyzing the production of phosphatidylinositol-4,5-bisphosphate (PIP2), a lipid that regulates fundamental cellular processes such as cytoskeletal organization, cell migration, and metabolic control [23]. PIPKIγ has emerged as a key player in cancer progression by promoting PIP2-dependent activation of the PI3K/AKT signaling pathway, a central regulator of cell survival and proliferation [24, 25]. Moreover, PIPKIγ contributes to cancer metastasis by generating PIP2 at focal adhesions, which facilitates the dynamic assembly and disassembly of these structures, thus promoting cancer cell migration and invasion [26–28]. Overexpression of PIPKIγ has been observed in BC and TNBC [24, 29], in which it is implicated in enhanced cell motility, invasiveness, and oncogenic growth [27, 28]. Beyond its roles in tumor biology, recent research has also linked PIPKIγ to age-related diseases such as osteoarthritis and degenerative disc disease, suggesting its broader significance in pathophysiology [30, 31]. Emerging evidence also suggests that PIPKIγ may be involved in DNA repair. It has been reported to affect oxaliplatin resistance in colorectal cancer [32]. Since platinum-based chemoresistance is often associated with alterations in DNA damage repair pathways [33], this raises the possibility that PIPKIγ may influence DDR regulation. Although its specific functions in DNA repair remain largely unexplored, this evidence provides a rationale for further investigation. Therefore, we hypothesize that PIPKIγ could modulate DSB repair, thereby influencing radiotherapy sensitivity in TNBC.

In this study, we aim to investigate how PIPKIγ regulates DNA damage repair and contributes to radiotherapy resistance in TNBC. We reveal the role of PIPKIγ in improving NHEJ repair efficiency through its interaction with LIG4, which contributes to increased radioresistance in TNBC. Specifically, PIPKIγ strengthens the interaction between LIG4 and XRCC4, a key regulator of LIG4 nuclear translocation, thereby facilitating LIG4’s nuclear localization and improving NHEJ repair efficiency. In vitro and in vivo functional assays show that PIPKIγ overexpression correlates with increased cell survival following radiation, whereas PIPKIγ knockout sensitizes TNBC cells to radiation, indicating its critical role in modulating radiotherapy response. Our findings not only identify a novel mechanism by which PIPKIγ promotes radiation resistance in TNBC, but also suggest that targeting PIPKIγ could offer a promising therapeutic strategy to overcome radiation resistance in TNBC, thereby potentially improving clinical outcomes for patients.

## Materials and methods

To ensure clarity and in consideration of word count limitations, detailed descriptions of the experimental procedures are provided in the Supplementary Materials and Methods.

### Cell culture

HCA2-hTERT cells, HCA2-I9a cells (a derivative of HCA2-hTERT containing a single NHEJ reporter cassette), HCA2-H15c cells (harboring an HR reporter cassette), CLZ3 cells (derived from the D4a human fibroblast line [34] and optimized for HR and NHEJ efficiency), HEK293T, and MDA-MB-231 cells were cultured in DMEM (KeyGen Biotech, Cat. # KGM12800) supplemented with 10% fetal bovine serum (FBS; Life Technologies, Cat. # 16000), 1% non-essential amino acids (NEAA; Gibco, Cat. # 11140-050), and 1% penicillin-streptomycin (Gibco, Cat. # 15140-122). SUM159PT cells were maintained in RPMI-1640 medium (KeyGen Biotech, Cat. # KGL1501-500) with the same supplements. All cultures were incubated at 37°C with 5% CO_2_. All cell lines tested negative for mycoplasma contamination.

### Statistical analysis

Statistical analyses were conducted using GraphPad Prism 10.0. Kaplan–Meier curves were used to assess group differences in prognosis. Cox proportional hazards models, both univariate and multivariate, evaluated the association between indicated parameters and outcomes. Statistical significance was set at *P* < 0.05, with the following representations: ns (*P* ≥ 0.05), (*P < 0.05), (**P < 0.01), (***P < 0.001), (****P < 0.0001).

## Results

### PIPKIγ enhances radioresistance in TNBC and promotes NHEJ independently of its canonical kinase activity

**Fig. 1:**
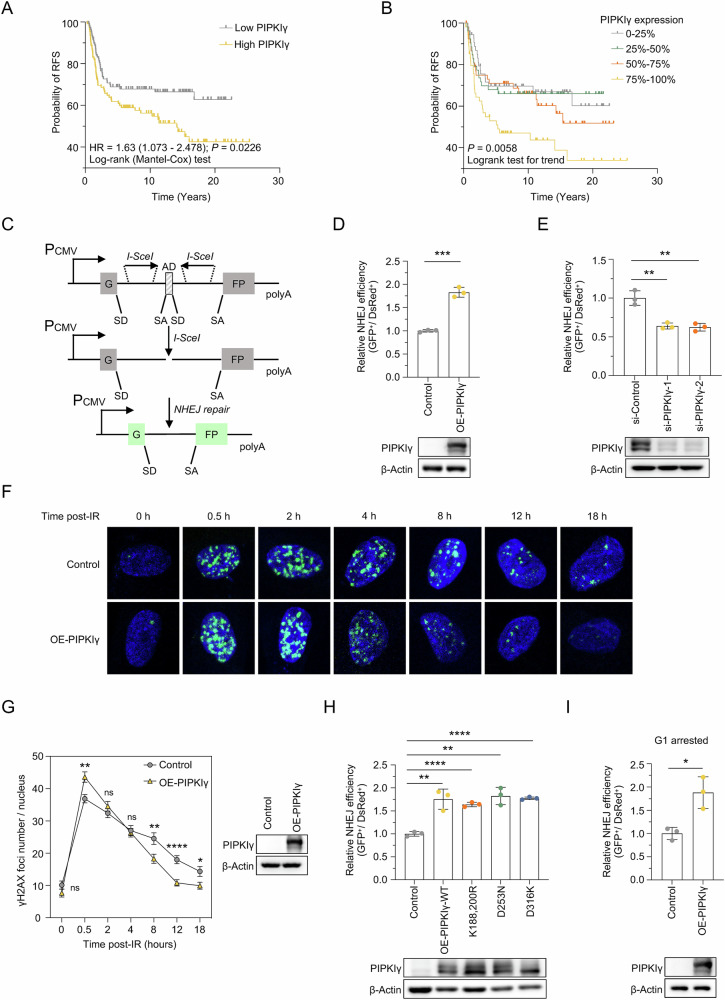
PIPKIγ enhances radioresistance in TNBC and promotes NHEJ independently of its canonical kinasse activity. **A** The association between median PIPKIγ expression and RFS probability in TNBC patients undergoing radiotherapy. **B** The association between PIPKIγ expression quartiles and RFS probability in TNBC patients undergoing radiotherapy. **C** Schematic Diagram of the Well-Established NHEJ (pEGFP-Pem1-Ad2) Reporter. The operational principles of this reporter system were detailed in Seluanov et al., 2004, PNAS. This NHEJ reporter allows the evaluation of both canonical c-NHEJ and alternative NHEJ alt-NHEJ efficiencies. **D** NHEJ efficiency in HCA2-I9a cells overexpressing PIPKIγ. Cells were transfected with I-SceI, DsRed2-N1, and PIPKIγ vectors. After 72 h, FACS analysis was performed. *n* = 3 per group. Western blot showing PIPKIγ overexpression is included. Data are shown as mean ± SD (Unpaired *t* test; ***P < 0.001). **E** NHEJ efficiency in HCA2-I9a cells with PIPKIγ knockdown. Western blot showing PIPKIγ knockdown is included. *n* = 3 per group. Data are shown as mean ± SD (Unpaired *t* test; **P < 0.01). **F**, **G** Impact of PIPKIγ overexpression on γH2AX foci clearance. HCA2-hTERT cells were transfected with control or PIPKIγ vector, treated with 2 Gy X-ray, and immunostained for γH2AX foci at various time points. Representative images are shown in (**F**), and the number of γH2AX foci per nucleus is quantified in (**G**). Data are shown as mean ± SEM (Unpaired *t* test; ns, *P* ≥ 0.05; *P < 0.05; **P < 0.01; ****P < 0.0001). **H** NHEJ efficiency in HCA2-I9a cells overexpressing wild-type or mutant PIPKIγ. Western blot showing PIPKIγ overexpression is included. *n* = 3 per group. Data are shown as mean ± SD (Unpaired *t* test; **P < 0.01; ****P < 0.0001). **I** c-NHEJ efficiency in G1-arrested HCA2-I9a cells overexpressing PIPKIγ. Confluent HCA2-I9a cells were transfected with I-SceI, DsRed2-N1, and PIPKIγ vectors. After 72 h, FACS analysis was conducted. Western blot showing PIPKIγ overexpression is included. *n* = 3 per group. Data are shown as mean ± SD (Unpaired *t* test; *P < 0.05).

**Fig. 2:**
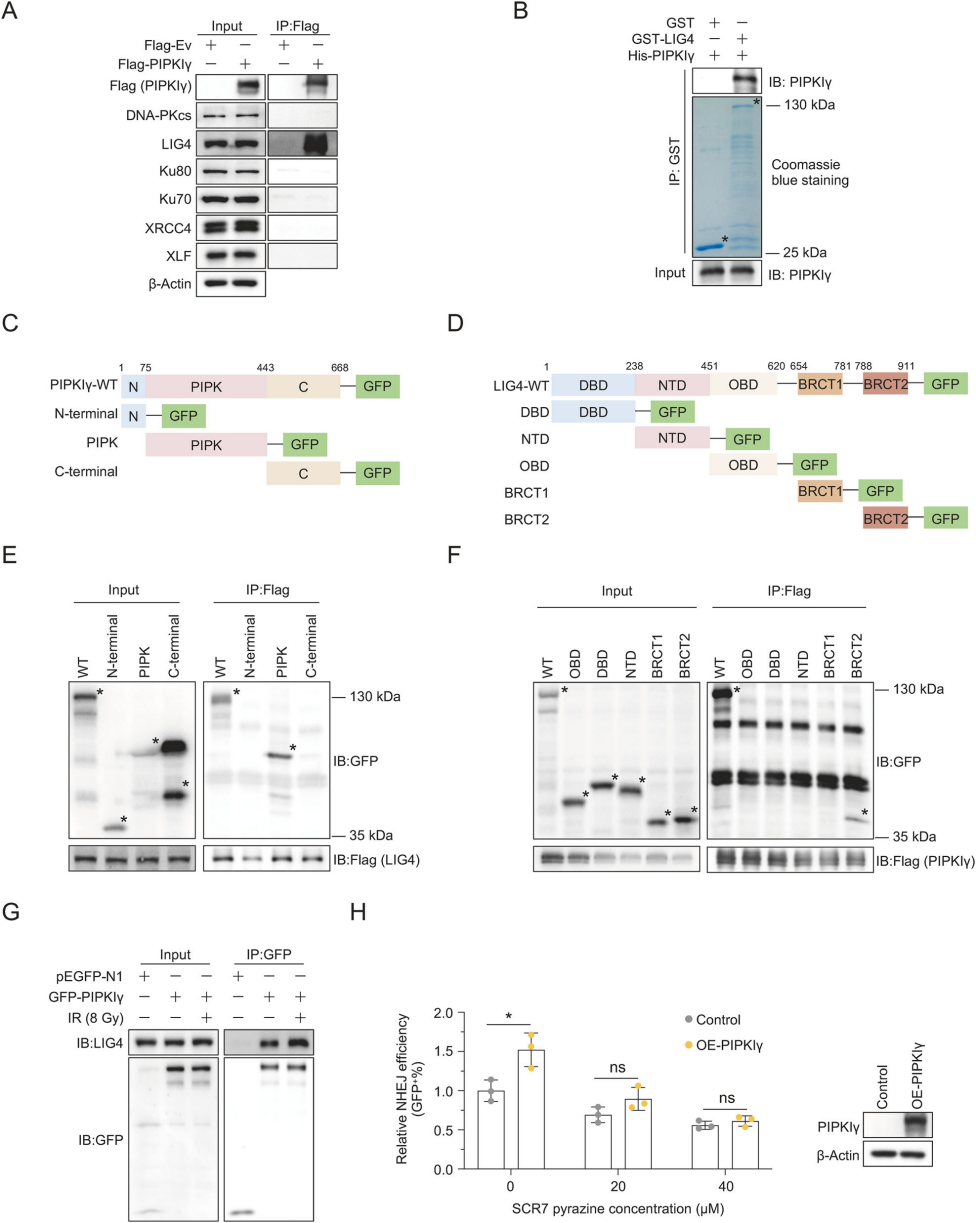
PIPKIγ directly interacts with LIG4 and enhances c-NHEJ in a LIG4-dependent manner. **A** Co-IP analysis of the interaction between PIPKIγ and key c-NHEJ proteins in HEK293T cells. **B** In vitro Co-IP analysis showing interaction between purified recombinant PIPKIγ and LIG4 proteins, followed by Western blotting. **C**, **E** Co-IP analysis demonstrating interaction between LIG4 and specific domains of PIPKIγ in HEK293T cells. The domain organization of PIPKIγ, as illustrated in (**C**), was based on Thapa et al., 2016, Trends Cancer. **D**, **F** Co-IP analysis showing interaction between PIPKIγ and specific domains of LIG4 in HEK293T cells. The domain organization of LIG4, as depicted in (**D**), was based on Kaminski et al., 2018, Nat Commun. **G** Co-IP analysis of the interaction between PIPKIγ and LIG4 in HEK293T cells exposed to 8 Gy X-ray radiation. **H** Analysis of c-NHEJ efficiency in G1-arrested CLZ3 cells overexpressing PIPKIγ, treated with or without SCR7 pyrazine. Confluent CLZ3 cells were induced with 0.5 μg/ml doxycycline to express I-SceI. After 72 h, FACS analysis was performed. Western blot showing PIPKIγ overexpression is included. *n* = 3 per group. Data are shown as mean ± SD (Unpaired *t* test; ns, *P* ≥ 0.05; *P < 0.05).

PIPKIγ is known for its elevated expression in BC and TNBC [24, 29], with established roles in driving proliferation, invasion, and metastasis in these cancers [26–28]. Given these findings, we aimed to explore the clinical relevance of PIPKIγ expression in the context of radiotherapy resistance. Here, we utilized the Molecular Taxonomy of Breast Cancer International Consortium (METABRIC) database to assess the association between PIPKIγ expression levels and recurrence-free survival (RFS) across three BC subtypes—HER2-enriched, luminal, and TNBC—among patients who received radiotherapy. Our analysis revealed that elevated PIPKIγ expression was associated with poorer RFS exclusively in the TNBC cohort that had undergone radiotherapy (Fig. 1A; *P* = 0.0226, HR = 1.63 (1.073–2.478)), whereas no significant association was observed in the HER2-enriched or luminal subtypes (Supplementary Fig. 1A, B; *P* > 0.05). Additionally, when stratified by quartiles, an expression-dependent correlation between PIPKIγ expression and declining RFS was observed in TNBC patients (Fig. 2B). Univariate Cox regression analysis further confirmed that PIPKIγ expression was significantly correlated with RFS in TNBC (Table 1; *P* = 0.036, HR = 1.681 (1.036–2.728)). Multivariate stepwise Cox regression analysis demonstrated that this association was independent of other clinicopathological factors (Table 2; *P* = 0.043, HR = 1.750 (1.018–3.009)). These findings suggest that PIPKIγ is likely to contribute to the progression of TNBC, with one of the potential mechanisms being the enhancement of resistance to radiotherapy.

**Table 1:** Univariate COX analysis for RFS in TNBC patients treated with radiotherapy from the METABRIC database.

Covariate	Hazard ratio (95% CI)	*P* value
PIPKIγ	1.681 (1.036–2.728)	0.036
Age	0.992 (0.974–1.011)	0.409
Lymph nodes examined positive		
NO	Reference	
YES	1.895 (1.148–3.129)	0.013
Tumor grade		
Grade1 + Grade2	Reference	
Grade3	1.822 (0.787–4.218)	0.161
Tumor stage		
Stage1 + Stage 2	Reference	
Stage3	2.903 (1.628–5.175)	< 0.001

**Table 2:** Munivariate COX analysis for RFS in TNBC patients treated with radiotherapy from the METABRIC database.

Covariate	Hazard ratio (95% CI)	*P* value
PIPKIγ	1.750 (1.018–3.009)	0.043
Tumor stage		
Stage1 + Stage 2	Reference	
Stage3	2.145 (1.142–4.031)	0.018

Since radiotherapy primarily exerts its cytotoxic effects through the induction of DSBs, the upregulation of DSB repair by NHEJ and HR in tumors confers resistance to radiation [14]. A previous study suggested a potential role of PIPKIγ in DNA repair, but its specific mechanism remains unclear [32]. Based on these observations, we hypothesize that PIPKIγ may activate DSB repair pathways, thereby contributing to radiotherapy resistance in TNBC.

To examine whether PIPKIγ regulates DSBs repair, we employed our well-established HCA2-H15c and HCA2-I9a cell lines that may separately analyze HR and NHEJ efficiency [35] (Fig. 1C and Supplementary Fig. 1C). We found that PIPKIγ overexpression promotes NHEJ repair but not HR directed repair (Fig. 1D and Supplementary Fig. 1D). Additionally, PIPKIγ knockdown reduced NHEJ repair efficiency (Fig. 1E). To further elucidate the role of PIPKIγ in DSBs repair, we performed immunostaining experiments to assess the dynamics of γH2AX foci clearance, a DSB marker, in PIPKIγ-overexpressing cells following X-ray treatment. Our findings revealed that cells overexpressing PIPKIγ enhanced clearance of γH2AX. Specifically, these cells showed a reduction in γH2AX foci at 8, 12, and 18 hours post-irradiation (Fig. 1F, G). These data indicate that PIPKIγ plays a role in the repair of DSBs.

As a kinase, PIPKIγ generates PIP2, which activates the PI3K/AKT pathway to boost tumor proliferation and integrin-mediated focal adhesions, thereby promoting invasion and migration [23]. We then conducted our DNA repair assay to examine whether its stimulatory effect is dependent on its enzymatic activity. Overexpressing three enzymatically inactive PIPKIγ mutants (K188/200 R, D253N, and D316K) [36–38] failed to abolish the stimulatoy effect on NHEJ (Fig. 1H), and inhibiting PIPKIγ kinase activity using its specific inhibitor UNC3230 [25, 39, 40] had no effect on NHEJ (Supplementary Fig. 2A). These results reveal that PIPKIγ enhances NHEJ independent of its canonical enzymatic activity.

The NHEJ pathway comprises two sub-pathways: the canonical NHEJ (c-NHEJ), characterized by higher accuracy and activity throughout the cell cycle, and the alternative NHEJ (alt-NHEJ), which is less precise, predominantly active during the S and G2 phases, and typically leads to mutations [41–45]. To ascertain which sub-pathway is promoted by PIPKIγ, we evaluated its impact on both c-NHEJ and alt-NHEJ separately.

Previous research indicates that the majority of DSBs in cells arrested in the G1 phase are repaired through the c-NHEJ pathway [46]. We therefore induced G1 arrest in the NHEJ-I9a reporter fibroblast cell line by culturing them to confluence. The arrest was confirmed by EdU assay, western blotting with an anti-Ki67 antibody, and the analysis of cell cycle distribution (Supplementary Fig. 2C-E). We found that c-NHEJ efficiency was significantly enhanced in G1-arrested NHEJ-I9a cells overexpressing PIPKIγ (Fig. 1I). Additionally, in actively proliferating cells, inhibition of PARP1 enzymatic activity to suppress alt-NHEJ did not diminish the PIPKIγ-mediated enhancement of NHEJ efficiency. (Supplementary Fig. 2B). Moreover, using a GFP-based reporter cassette, which has 8 nucleotides of microhomology flanking the I-SceI recognition sites to enable the quantification of alt-NHEJ activity [47] (Supplementary Fig. 3A), we found that overexpressing PIPKIγ did not enhance alt-NHEJ (Supplementary Fig. 3B). Taken together, these data indicate that PIPKIγ stimulates c-NHEJ repair rather than alt-NHEJ.

### PIPKIγ directly interacts with LIG4 and enhances c-NHEJ in a LIG4-dependent manner

To elucidate the mechanism by which PIPKIγ enhances c-NHEJ, we first performed Western blot analysis to examine whether PIPKIγ affects the expression of c-NHEJ factors such as DNA-PKcs, Ku70, Ku80, LIG4, XLF, and XRCC4. The results indicate that overexpression or knockout of PIPKIγ in MDA-MB-231 cells did not affect the protein levels of these c-NHEJ factors (Supplementary Fig. 3C, D). Next, we conducted co-immunoprecipitation (co-IP) experiments to assess interactions with key c-NHEJ factors. Our results revealed an interaction between PIPKIγ and LIG4, with no detectable interactions with the other c-NHEJ factors (Fig. 2A). The in vitro co-IP assay further confirmed that PIPKIγ and LIG4 directly interacted with each other (Fig. 2B). Next, we characterized which domains of the two factors mediate their interaction. By performing co-IP experiments with vectors expressing full-length and truncated PIPKIγ or LIG4, we found that the PIPK domain of PIPKIγ mediated its interaction with LIG4 (Fig. 2C, E), while the BRCT2 domain of LIG4 is vital to its interaction with PIPKIγ (Fig. 2D, F).

To ascertain if the interaction between PIPKIγ and LIG4 is important to DNA repair, we performed co-IP experiments in HEK293T cells following X-ray exposure. The findings indicated that the interaction between PIPKIγ and LIG4 is indeed strengthened in response to DNA damage (Fig. 2G), suggesting that the formation of the PIPKIγ-LIG4 complex is crucial for DNA repair. To further validate our hypothesis, we investigated the alterations in NHEJ activity upon overexpression of PIPKIγ, with or without treatment with SCR7 pyrazine, an LIG4 inhibitor, in CLZ3 cells harboring an HR-NHEJ reporter cassette (Supplementary Fig. 3E, F) [34, 48]. The DNA repair assay revealed that inhibiting LIG4 enzymatic activity reduced the stimulatory effect of PIPKIγ on NHEJ (Fig. 2H), indiating that PIPKIγ promotes NHEJ through LIG4.

In conclusion, these results demonstrate that PIPKIγ directly interacts with LIG4 and promotes NHEJ through LIG4.

### PIPKIγ Promotes the nuclear translocation of LIG4 through strengthening the LIG4-XRCC4 Interaction

**Fig. 3:**
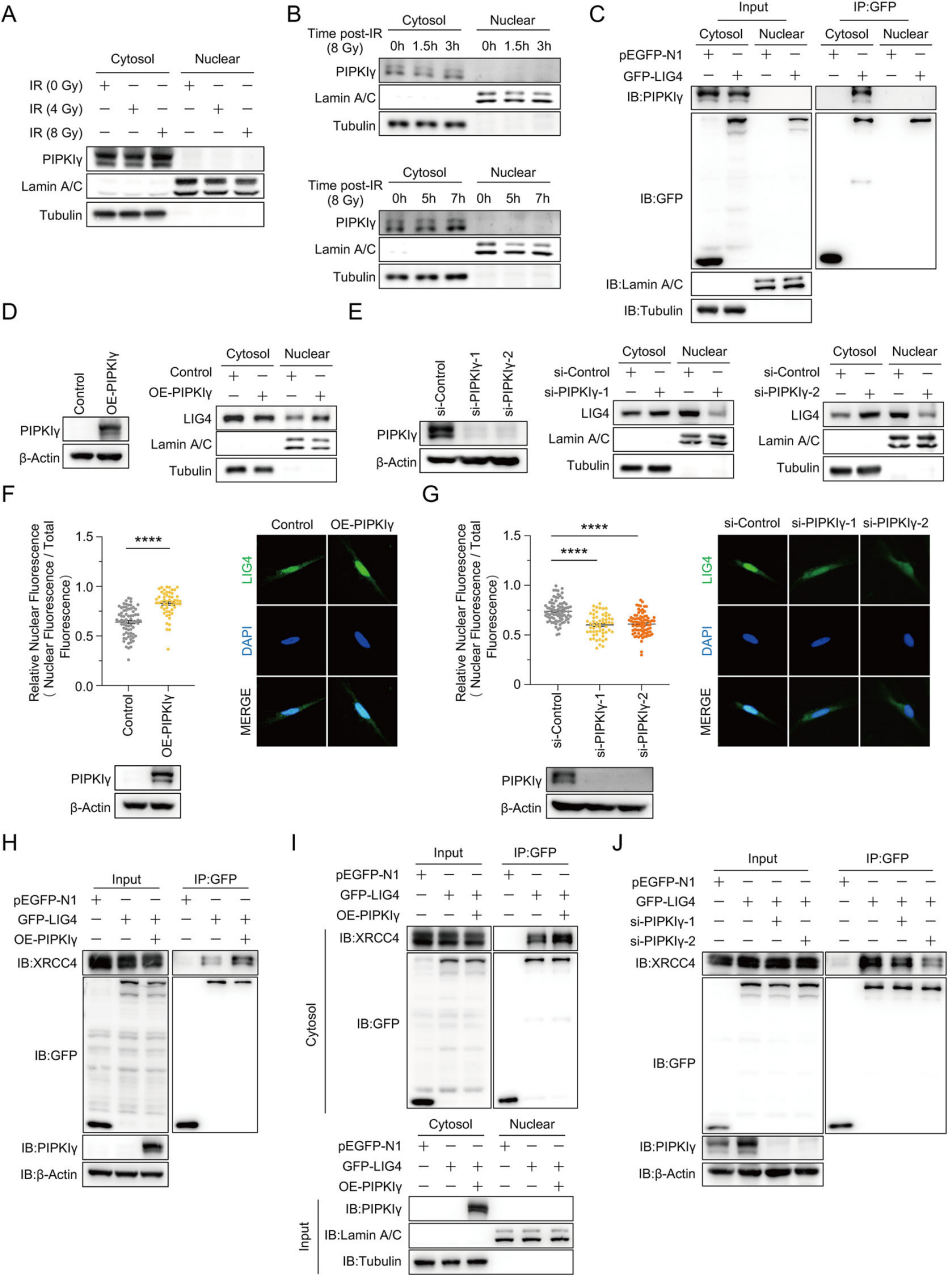
PIPKIγ Promotes the nuclear translocation of LIG4 through strengthening the LIG4-XRCC4 Interaction. **A** Western blot analysis of PIPKIγ protein levels in the cytoplasmic and nuclear fractions of HEK293T cells, with and without X-ray exposure. Tubulin and Lamin A/C were used as markers for cytoplasmic and nuclear fractions, respectively. **B** Western blot analysis of PIPKIγ in the cytoplasmic and nuclear fractions of MDA-MB-231 cells at various time points post-X-ray radiation. **C** Co-IP analysis showing the interaction between PIPKIγ and LIG4 in the cytoplasmic and nuclear fractions. **D** Western blot analysis of LIG4 levels in the cytoplasmic and nuclear fractions of MDA-MB-231 cells overexpressing PIPKIγ. Confirmation of PIPKIγ overexpression is included. **E** Western blot analysis of LIG4 in the cytoplasmic and nuclear fractions of MDA-MB-231 cells with PIPKIγ knockdown. Confirmation of PIPKIγ knockdown is included. **F** Immunofluorescent staining of LIG4 in the cytoplasmic and nuclear fractions of MDA-MB-231 cells overexpressing PIPKIγ. LIG4 distribution was analyzed using relative nuclear fluorescence (Nuclear Fluorescence/Total Fluorescence). Confirmation of PIPKIγ overexpression and representative images of LIG4 distribution are shown. Data are shown as mean ± SEM (Unpaired *t* test; ****P < 0.0001). **G** Immunofluorescent staining of LIG4 in the cytoplasmic and nuclear fractions of MDA-MB-231 cells with PIPKIγ knockdown. Confirmation of PIPKIγ knockdown and representative images of LIG4 distribution are shown. Data are shown as mean ± SEM (Unpaired *t* test; ****P < 0.0001). **H** Co-IP analysis demonstrating strengthened interaction between XRCC4 and LIG4 in HEK293T cells overexpressing PIPKIγ. **I** Co-IP analysis confirming that the strengthened interaction between XRCC4 and LIG4 occurs in the cytoplasmic fraction in HEK293T cells overexpressing PIPKIγ. **J** Co-IP analysis showing weakened interaction between XRCC4 and LIG4 in HEK293T cells with PIPKIγ knockdown.

Given that LIG4 functions in NHEJ within the nucleus, we aimed to investigate whether the interaction between PIPKIγ and LIG4 occurs in the nucleus. We conducted subcellular fractionation experiments followed by Western blot analysis on HEK293T and MDA-MB-231 cells exposed to X-ray irradiation. The results indicated that PIPKIγ is predominantly cytoplasmic, with no evidence of nuclear translocation even upon DNA damage induction (Fig. 3A, B). Subcellular fractionation followed by co-IP analysis confirmed that the interaction between PIPKIγ and LIG4 occurs in the cytosol rather than in the nucleus (Fig. 3C). These findings suggest that PIPKIγ may exert its regulatory effects on c-NHEJ in an indirect manner.

It is a common phenomenon for DNA repair factors or regulators to directly translocate to the nucleus to engage in DNA repair processes [49–52]. LIG4 is well-known for its nuclear localization, playing an indispensable role in the final ligation step of NHEJ repair [17], but it has also been detected in the cytoplasm [53–55]. We therefore hypothesize that PIPKIγ might facilitate the nuclear translocation of LIG4. Indeed, our subcellular fractionation analysis demonstrated that LIG4 was accumulated in the nuclear fractions of PIPKIγ-overexpressing cells, and less prevalent in the nucleus of PIPKIγ-knockdown cells compared to the control, indicating that PIPKIγ promotes LIG4 nuclear translocation (Fig. 3D, E). Furthermore, immunostaining experiments demonstrated that cells overexpressing PIPKIγ exhibited increased relative nuclear fluorescence intensity of LIG4, while PIPKIγ-knockdown cells displayed a reduced relative nuclear fluorescence intensity compared to the control group (Fig. 3F, G). Taken together, these data indicate that PIPKIγ promotes NHEJ repair by stimulating the LIG4 nuclear translocation.

Previous studies have shown that XRCC4 governs the nuclear import and distribution of LIG4, and that the LIG4-XRCC4 interaction in the cytoplasm is essential for LIG4 nuclear translocation [53, 55]. Based on this, we hypothesized that PIPKIγ facilitates LIG4 nuclear translocation by strengthening its interaction with XRCC4. Consistent with this hypothesis, co-IP experiments revealed that overexpressing PIPKIγ significantly enhanced the LIG4-XRCC4 interaction (Fig. 3H), while subcellular fractionation co-IP analysis demonstrated that this interaction occurs in the cytoplasm (Fig. 3I). Conversely, knockdown of PIPKIγ weakened the LIG4-XRCC4 interaction (Fig. 3J).

In summary, our findings demonstrate that PIPKIγ promotes the nuclear translocation of LIG4 by strengthening the LIG4-XRCC4 interaction, thereby facilitating efficient NHEJ repair.

### PIPKIγ promotes genomic stability and enhances radiation resistance in TNBC cells

Since NHEJ is crucial for repairing ionizing radiation-induced DSBs to stabilize genomes, thereby avoiding cellular senescence or cell death [56–59], we explored whether PIPKIγ enhances radiation resistance by promoting c-NHEJ in TNBC cells.

To examine the role of PIPKIγ in radiotherapy resistance, we selected MDA-MB-231 and SUM159PT, two TNBC cell lines commonly used in studies of radioresistance. Western blot analysis confirmed that both cell lines exhibit higher PIPKIγ expression compared to the non-tumorigenic mammary epithelial cell line MCF-10A (Supplementary Fig. 4A), thereby supporting their selection for subsequent functional experiments.

**Fig. 4:**
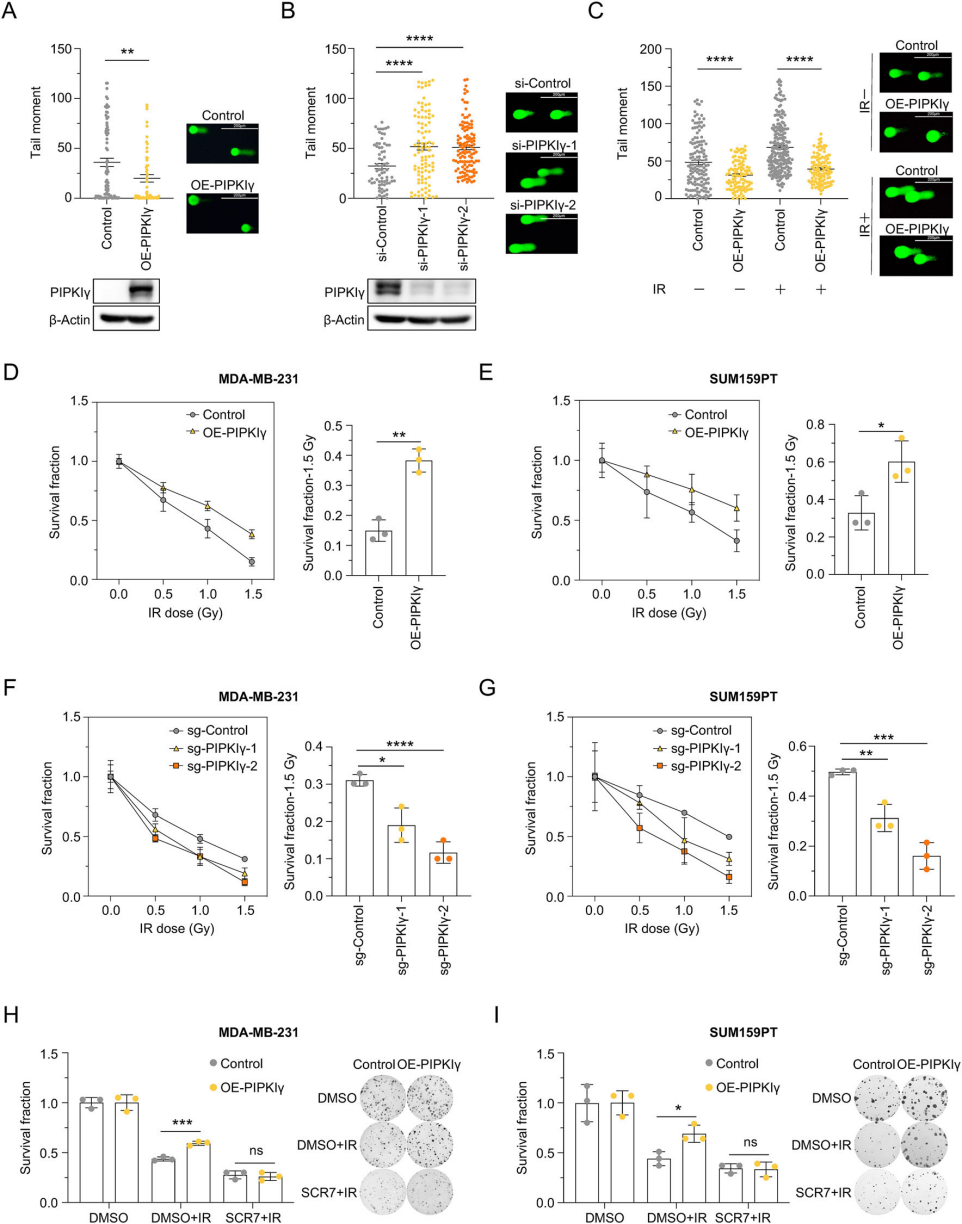
PIPKIγ promotes genomic stability and enhances radiation resistance in TNBC cells. **A** Genomic instability analysis in control and PIPKIγ-overexpressing MDA-MB-231 cells using the comet assay. The tail moment was used as a measure of genomic instability, with at least 50 cells analyzed via CometScore software. Representative comet assay images of PIPKIγ-overexpressing MDA-MB-231 cells are shown. Data are shown as mean ± SEM (Unpaired *t* test; **P < 0.01). **B** Genomic instability analysis in control and PIPKIγ-knockdown MDA-MB-231 cells using the comet assay. Representative comet assay images of PIPKIγ-knockdown MDA-MB-231 cells are shown. Data are shown as mean ± SEM (Unpaired *t* test; ****P < 0.0001). **C** Genomic instability analysis in PIPKIγ-overexpressing MDA-MB-231 cells treated with or without IR (4 Gy) using the comet assay. Representative comet assay images of PIPKIγ-overexpressing MDA-MB-231 cells, with or without IR treatment, are shown. Data are shown as mean ± SEM (Unpaired *t* test; ****P < 0.0001). **D**, **E** Clonogenic assays assessing the survival of PIPKIγ-overexpressing MDA-MB-231 and SUM159PT cells treated with different doses of IR. *n* = 3 per group. Data are shown as mean ± SD (Unpaired *t* test; *P < 0.05; **P < 0.01). **F**, **G** Clonogenic assays assessing the survival of PIPKIγ-knockout MDA-MB-231 and SUM159PT cells treated with different doses of IR. *n* = 3 per group. Data are shown as mean ± SD (Unpaired *t* test; *P < 0.05; **P < 0.01; ***P < 0.001; ****P < 0.0001). **H**, **I** Clonogenic assays assessing the survival of PIPKIγ-overexpressing MDA-MB-231 and SUM159PT cells treated with IR (1 Gy), with or without SCR7 pyrazine (80 μM for MDA-MB-231, 120 μM for SUM159PT). *n* = 3 per group. Data are shown as mean ± SD (Unpaired *t* test; *P < 0.05; ***P < 0.001).

Subsequently, Comet assay revealed that overexpression of PIPKIγ in MDA-MB-231 cells increased genomic stability, in both the X-ray untreated and X-ray irradiated cells, as measured by tail moment (Fig. 4A, C). Under X-ray irradiation, the increase in tail moment was notably attenuated in PIPKIγ-overexpressing cells compared with control cells, suggesting that PIPKIγ helps preserve genomic stability upon DSBs (Supplementary Fig. 4B). Conversely, depleting PIPKIγ impaired genomic stability in MDA-MB-231 cells (Fig. 4B). This was further supported by the percentage of DNA in the tail, another widely-accepted parameter for genomic stability [60] (Supplementary Fig. 4C–E).

In addition, we conducted clonogenic assay in MDA-MB-231 and SUM159PT cells. We observed that PIPKIγ-overexpressing cells exhibited heightened resistance to radiation, while PIPKIγ knockdown sensitized cells to X-ray-induced DNA damage (Fig. 4D–G and Supplementary Fig. 4F-I). Moreover, in PIPKIγ-overexpressing cells treated with the LIG4 inhibitor SCR7 pyrazine, the resistance to X-rays was abolished, indicating that PIPKIγ mediates radiation resistance via LIG4 in TNBC cells (Fig. 4H, I).

Together, these findings demonstrate that PIPKIγ promotes genomic stability and enhances radiation resistance in TNBC cells.

### PIPKIγ enhances radiation resistance in vivo

**Fig. 5:**
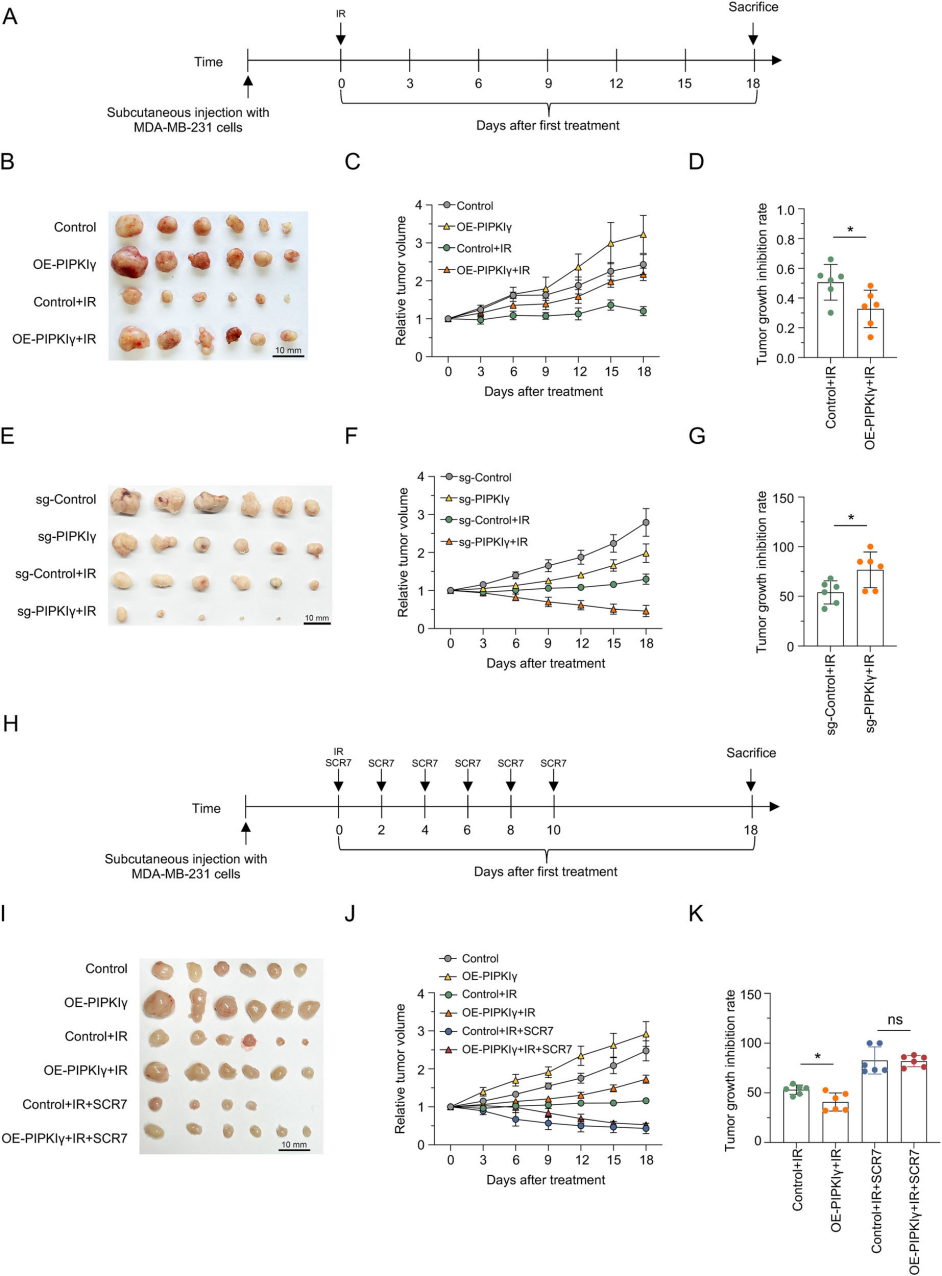
PIPKIγ Enhances Radiation Resistance In Vivo*.* **A** Schematic of the experimental design to evaluate the effect of PIPKIγ overexpression or knockout on radiotherapy sensitivity using a xenograft model in nude mice. *n* = 6 per group. **B** Representative images of tumor growth in nude mice across four experimental groups: control, PIPKIγ overexpression, control with IR, and PIPKIγ overexpression with IR. **C** Relative tumor volume comparison among four experimental groups: control, PIPKIγ overexpression, control with IR, and PIPKIγ overexpression with IR. **D** Tumor growth inhibition rate in control versus PIPKIγ-overexpressing groups with IR treatment. Data are shown as mean ± SD (Unpaired *t* test; *P < 0.05). **E** Representative images of tumor growth in nude mice across four experimental groups: sg-control, sg-PIPKIγ, sg-control with IR, and sg-PIPKIγ with IR. **F** Relative tumor volume comparison among four experimental groups: sg-control, sg-PIPKIγ, sg-control with IR, and sg-PIPKIγ with IR. **G** Tumor growth inhibition rate in sg-control versus sg-PIPKIγ groups with IR treatment. Data are shown as mean ± SD (Unpaired *t* test; *P < 0.05). **H** Schematic of the experimental design to evaluate whether SCR7 pyrazine treatment overcomes the radiotherapy resistance induced by PIPKIγ overexpression in a xenograft model using nude mice. *n* = 6 per group. **I** Representative images of tumor growth in nude mice across six experimental groups: control, PIPKIγ overexpression, control with IR, PIPKIγ overexpression with IR, control with IR and SCR7 pyrazine treatment, and PIPKIγ overexpression with IR and SCR7 pyrazine treatment. **J** Relative tumor volume comparison among six experimental groups: control, PIPKIγ overexpression, control with IR, PIPKIγ overexpression with IR, control with IR and SCR7 pyrazine treatment, and PIPKIγ overexpression with IR and SCR7 pyrazine treatment. **K** Tumor growth inhibition rate comparison among four experimental groups: control with IR, PIPKIγ overexpression with IR, control with IR and SCR7 pyrazine treatment, and PIPKIγ overexpression with IR and SCR7 pyrazine treatment. Data are shown as mean ± SD (Unpaired *t* test; *P < 0.05).

To determine if PIPKIγ is a viable target for overcoming radioresistance, we established MDA-MB-231 xenografts in nude mice and evaluated the impact of PIPKIγ on in vivo radioresistance. Once tumors were palpable, they were precisely irradiated with X-rays while other regions were protected by lead plates (Fig. 5A). As anticipated, PIPKIγ-overexpressing tumors displayed a diminished response to X-ray irradiation compared to controls (Fig. 5B–D). Consistently, PIPKIγ-knockout tumors showed an enhanced response (Fig. 5E–G). These findings indicate that PIPKIγ contributes to radiation resistance in TNBC xenografts.

Furthermore, we performed epistasis assays to explore whether the function of PIPKIγ in radiation resistance is LIG4-mediated in vivo (Fig. 5H). The resistance to X-rays in PIPKIγ-overexpressing tumors was compromised by SCR7 pyrazine treatment, suggesting that PIPKIγ-mediated promotion of radiation resistance is LIG4-dependent in xenografts (Fig. 5I–K).

Altogether, these results establish that PIPKIγ enhances radiation resistance in TNBC in vivo.

## Discussion

In this study, we delved into the roles of PIPKIγ beyond its recognized function in phosphoinositide metabolism and uncovered its significant involvement in DNA damage repair, thereby unveiling a novel mechanism in cancer cell biology (Supplementary Fig. 5). We found that PIPKIγ regulates the nuclear translocation of LIG4, a core component of the NHEJ pathway, highlighting an unexpected nuclear function for this traditionally cytoplasmic protein. This nuclear regulatory function provides a mechanistic link between cytoplasmic signaling and genomic maintenance. Previously, PIPKIγ has been linked to the regulation of the cytoskeleton and key signaling pathways, including the PI3K/AKT pathway [23]. Our discovery that PIPKIγ influences the repair of DSBs expands the known functions of PIPKIγ and identifies it as a pivotal player in tumor cell survival post-radiotherapy. This shift from cytoplasmic signal modulation to nuclear DNA repair control underscores the biological versatility of PIPKIγ and its emerging relevance in therapy resistance. Targeting the PIPKIγ-LIG4 axis could significantly affect the efficacy of radiotherapy, especially in cancer types that heavily depend on robust DNA repair mechanisms.

LIG4 is a critical enzyme responsible for the final ligation step of the NHEJ pathway. Its nuclear localization is essential for DNA repair; however, the mechanism regulating LIG4 nuclear translocation remains unclear. In tumor cells, the correct localization of LIG4 is critical for maintaining genomic integrity, especially following exposure to DNA-damaging agents such as radiation. Disruption of the XRCC4-LIG4 complex can lead to LIG4 mis-localization, resulting in its retention in the cytoplasm or preventing its recruitment to chromatin-bound DNA lesions [53–55]. This creates an opportunity to explore the regulatory factors that control LIG4 localization and activity in tumor cells. Targeting these regulatory pathways could enhance the sensitivity of tumor cells to DNA-damaging therapies by impairing effective DNA repair.

Our investigation reveals that PIPKIγ is a key regulator of the XRCC4–LIG4 interaction, which is essential for LIG4 nuclear localization and efficient DNA repair via the NHEJ pathway. Overexpression of PIPKIγ strengthens the XRCC4–LIG4 complex, whereas its knockdown disrupts this interaction and impairs LIG4 nuclear import. These findings support a novel regulatory mechanism whereby PIPKIγ stabilizes the XRCC4–LIG4 complex to facilitate LIG4’s translocation into the nucleus and recruitment to chromatin-bound DNA lesions, thereby preserving genomic integrity.

Post-translational modifications, including phosphorylation and ubiquitination, may further modulate the nuclear-cytoplasmic distribution of LIG4, imposing an additional layer of regulation on this critical repair pathway. The identification of these regulatory factors could potentially uncover new therapeutic targets to enhance the effectiveness of radiotherapy in cancers with radioresistance. Our findings represent the first evidence linking PIPKIγ to the regulation of LIG4 nuclear import via XRCC4 stabilization.

Interestingly, PIPKIγ has been shown to regulate nuclear signaling in other contexts—for example, by promoting the nuclear import of β-catenin through phosphorylation at Ser552 and Ser675 [61]. This suggests that PIPKIγ might similarly influence LIG4 nuclear entry via post-translational modifications, potentially by modulating LIG4 phosphorylation and stabilizing its association with XRCC4. Previous studies have demonstrated that phosphorylation is pivotal for controlling the nuclear localization of DNA repair proteins [62, 63]. Future work should investigate whether PIPKIγ regulates the XRCC4–LIG4 interaction and LIG4 nuclear import through such post-translational mechanisms, thereby deepening our understanding of its role in the NHEJ pathway.

Regulation of the nuclear translocation of DNA repair proteins plays a critical role in cancer therapy, as it enables cancer cells to better withstand genotoxic stress from radiotherapy or chemotherapy, ultimately contributing to treatment resistance. Targeting the nuclear transport pathways of these repair proteins could present a therapeutic strategy to increase cancer cell sensitivity to DNA damage. Moreover, the nuclear localization of repair proteins could serve as a predictive molecular marker for treatment response, informing personalized treatment strategies. Disrupting these mechanisms could reduce cancer cell survival, inhibit recurrence, and improve therapeutic outcomes.

In conclusion, our research elucidates a novel function of PIPKIγ in modulating LIG4 to enhance NHEJ repair and promote radiotherapy resistance in TNBC. These findings deepen our understanding of the role of PIPKIγ in DNA repair and suggest that targeting the PIPKIγ-LIG4 axis could be a novel therapeutic approach to enhance treatment efficacy, particularly for TNBC patients undergoing radiotherapy.

## Supplementary information

Supplementary Figure

Supplementary Materials and Methods

Original Western Blots

## Data Availability

All data generated or analyzed in this study are included in the main manuscript and supplementary files. The original Western blot images are provided in the supplementary files. This study incorporated data from the METABRIC (Molecular Taxonomy of Breast Cancer International Consortium) database, which is publicly available through the cBioPortal (https://www.cbioportal.org/study/summary?id=brca_metabric).
