# Supplementary material for: PIPKIγ promotes non-homologous end joining through LIG4 to enhance radiotherapy resistance in triple-negative breast cancer
Source: Cell Death Dis. 2025 Jul 31;16(1):578. doi: 10.1038/s41419-025-07894-5 (PMC12314031; doi:10.1038/s41419-025-07894-5)
Supplement: Supplementary file 1 — Supplementary Figure [file 41419_2025_7894_MOESM1_ESM.pdf]

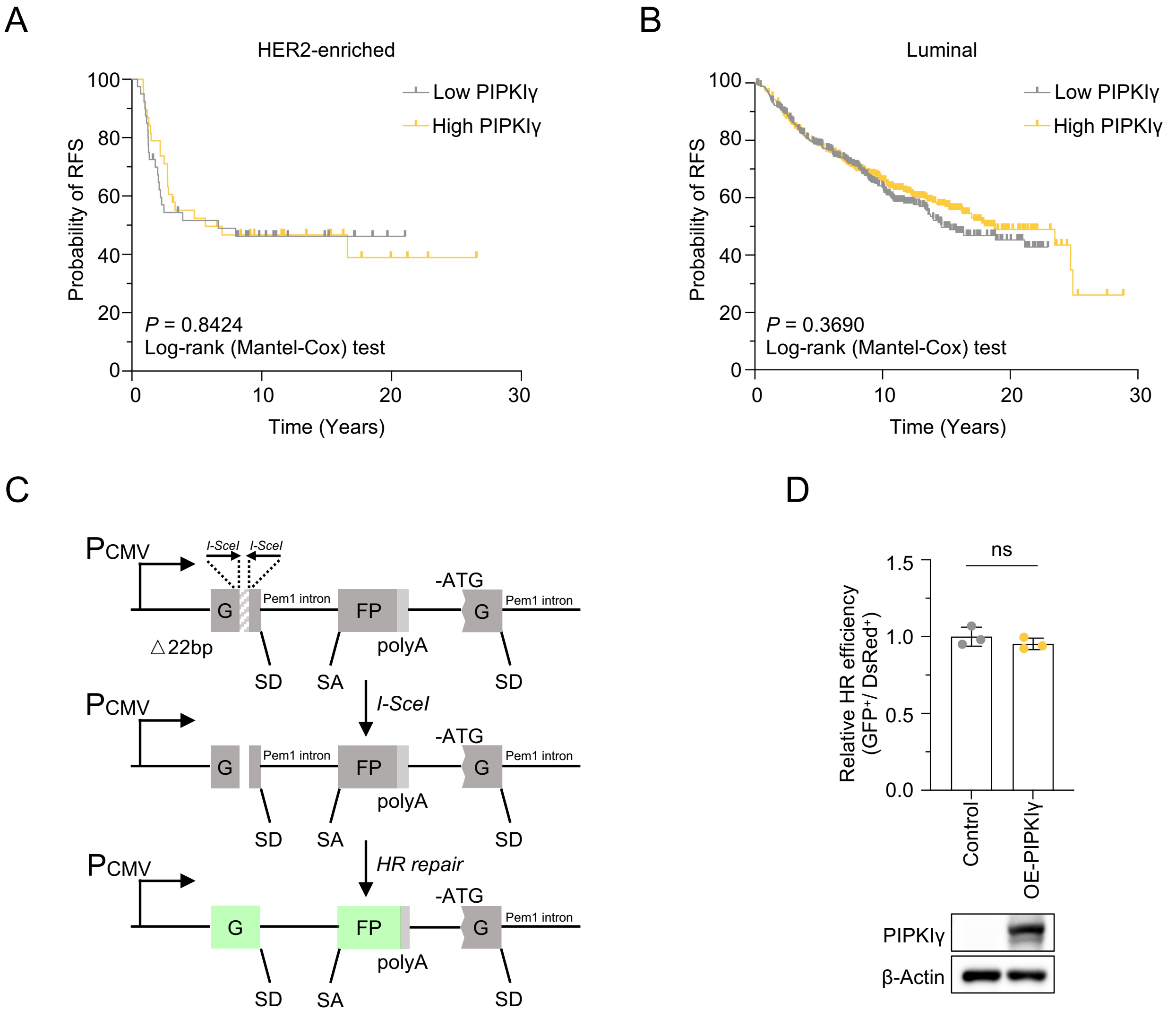

Supplementary Figure 1. (A) The association between median PIPKIγ expression and RFS probability in HER2-enriched breast cancer patients undergoing radiotherapy. (B) The association between median PIPKIγ expression and RFS probability in luminal breast cancer patients undergoing radiotherapy. (C) Schematic diagram of the well-established HR reporter. The mechanism of the reporter is described in Mao et al., 2007, PNAS. (D) HR efficiency in HCA2-H15c cells overexpressing PIPKIγ. n = 3 per group. Data are shown as mean ± SD (Unpaired t test; ns, $P \geq 0.05$).

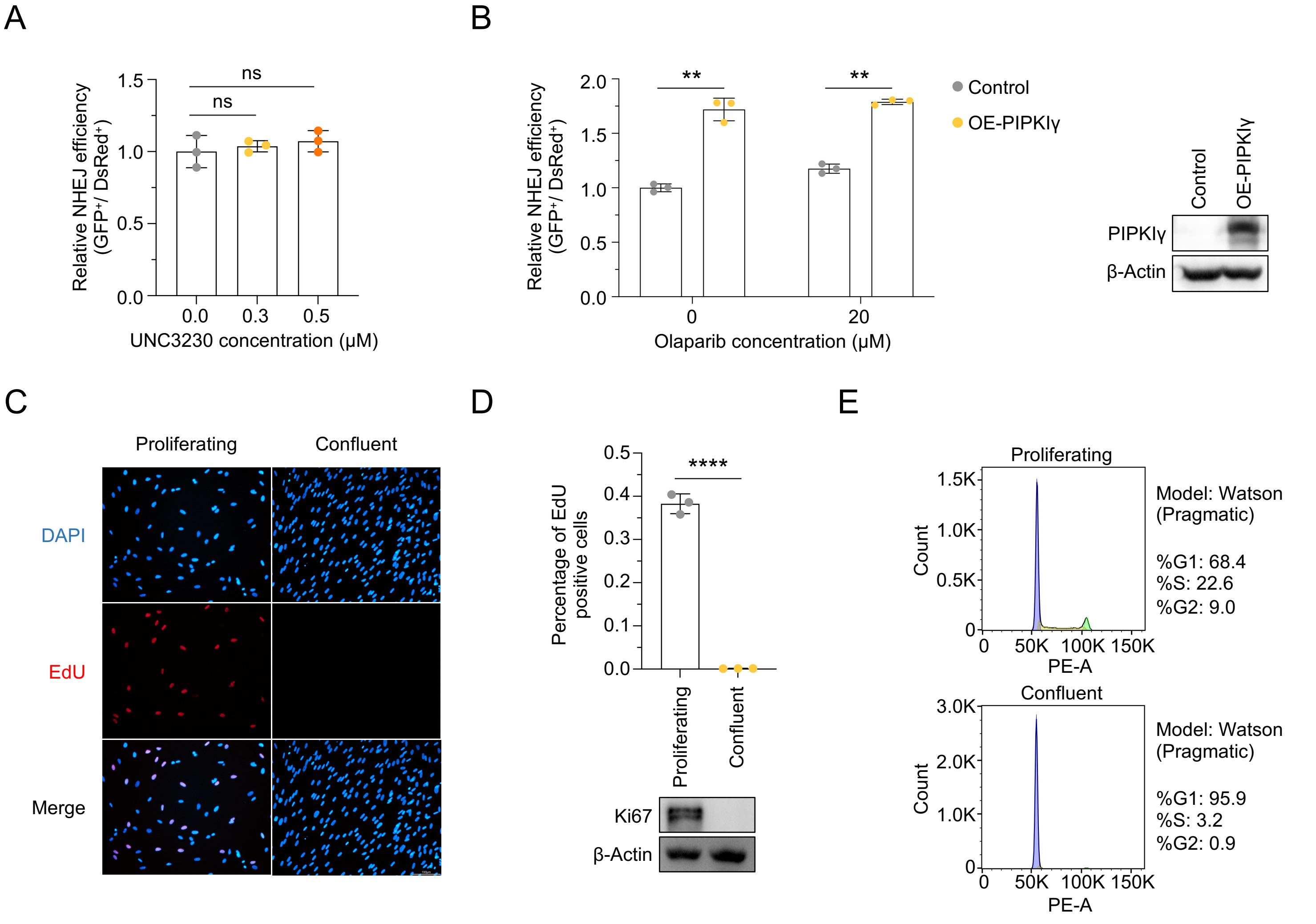

Supplementary Figure 2. (A) NHEJ efficiency in HCA2-I9a cells treated with various concentrations of UNC3230. n = 3 per group. Data are shown as mean ± SD (Unpaired t test; ns, P ≥ 0.05). (B) NHEJ efficiency in HCA2-I9a cells overexpressing PIPKIγ, with or without olaparib treatment. Western blot showing PIPKIγ overexpression is included. n = 3 per group. Data are shown as mean ± SD (Unpaired t test; **P < 0.01). (C) Representative images from the EdU assay showing proliferating and confluent HCA2-hTERT cells. Scale bar, 100 μm. (D) Analysis of the proportion of EdU-positive cells in proliferating and confluent HCA2-hTERT cells (top). Western blot analysis of the expression of the proliferation marker Ki67 in proliferating and confluent HCA2-hTERT cells (bottom). n = 3 per group. Data are shown as mean ± SD (Unpaired t test; ****P < 0.0001). (E) Cell cycle distribution in proliferating and confluent HCA2-hTERT cells.

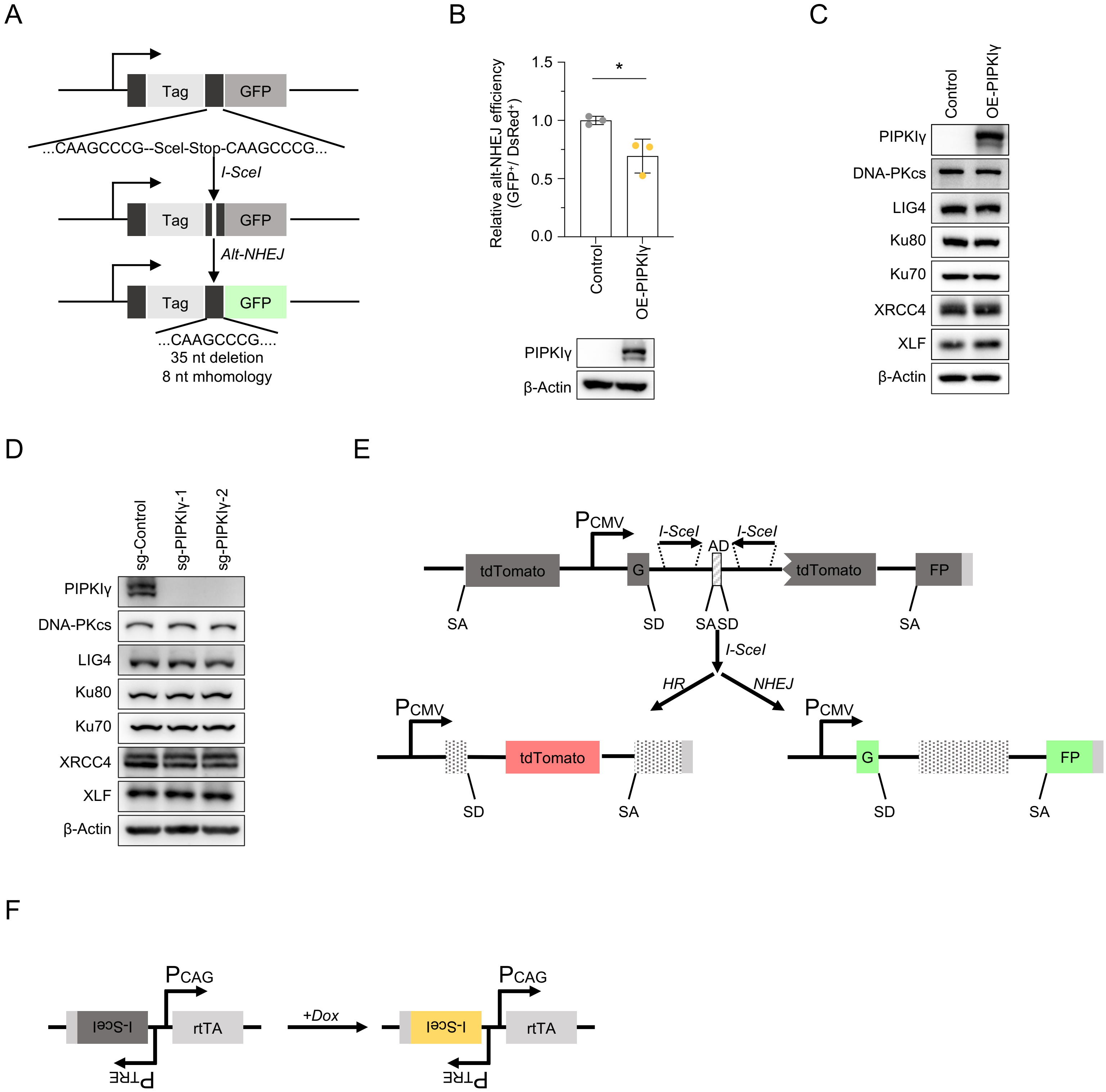

Supplementary Figure 3. (A) Schematic diagram of the EJ2-GFP reporter used to analyze alt-NHEJ efficiency. The mechanism for the reporter is detailed in Bennardo et al., 2008, PLoS Genet. Briefly, the reporter contains two 8 nt microhomology regions flanking the I-SceI recognition sites, allowing the analysis of alt-NHEJ efficiency. (B) Alt-NHEJ efficiency in HCA2-hTERT cells overexpressing PIPKIγ. Western blot showing PIPKIγ overexpression is included. n = 3 per group. Data are shown as mean ± SD (Unpaired t test; *P < 0.05). (C) Western blot analysis of key c-NHEJ proteins in MDA-MB-231 cells overexpressing PIPKIγ. (D) Western blot analysis of key c-NHEJ proteins in PIPKIγ-knockout MDA-MB-231 cells. (E) Diagram of the HR-NHEJ dual fluorescent reporter (Chen et al., 2019, Nucleic Acids Res). (F) Schematic diagram of the doxycycline-inducible I-SceI expression vector (Zhang et al., 2020, eLife).

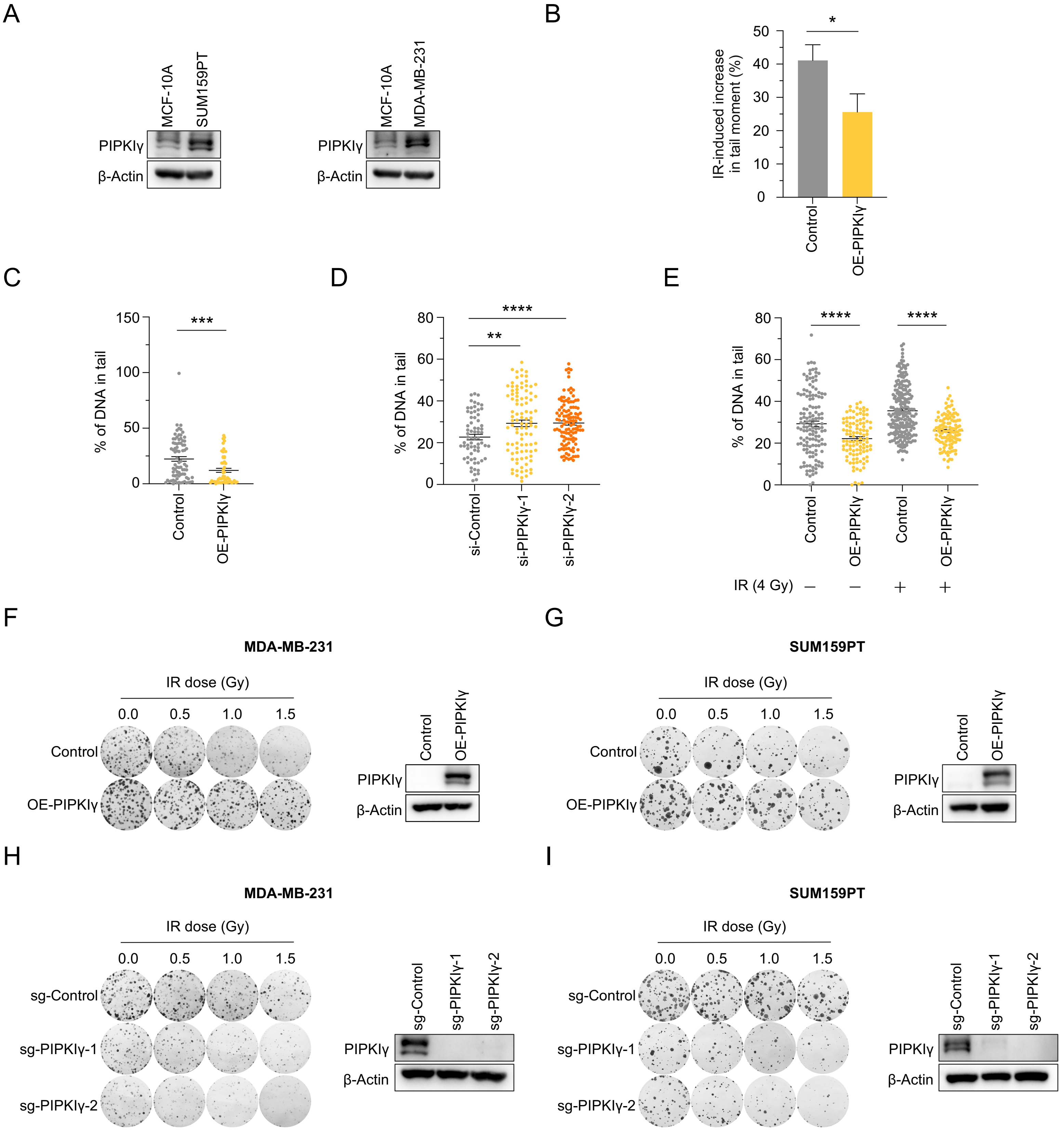

Supplementary Figure 4. (A) Western blot analysis of PIPKIγ expression in MCF-10A cells compared with MDA-MB-231 or SUMPT159 cells. (B) Quantification of IR-induced tail moment increase in control and PIPKIγ-overexpressing MDA-MB-231 cells. Data are shown as mean ± SEM (Unpaired t test; $^{*}P < 0.05$). (C) Analysis of the percentage of DNA in the tail of PIPKIγ-overexpressing MDA-MB-231 cells as determined by the comet assay. Data are shown as mean ± SEM (Unpaired t test; $^{***}P < 0.001$). (D) Analysis of the percentage of DNA in the tail of PIPKIγ-knockdown MDA-MB-231 cells as determined by the comet assay. Data are shown as mean ± SEM (Unpaired t test; $^{**}P < 0.01$; $^{****}P < 0.0001$). (E) Analysis of the percentage of DNA in the tail of PIPKIγ-overexpressing MDA-MB-231 cells, treated with or without IR, as determined by the comet assay. Data are shown as mean ± SEM (Unpaired t test; $^{****}P < 0.0001$). (F, G) Representative images of clonogenic assays assessing the survival of PIPKIγ-overexpressing MDA-MB-231 and SUM159PT cells treated with different doses of IR. (H, I) Representative images of clonogenic assays assessing the survival of PIPKIγ-knockout MDA-MB-231 and SUM159PT cells treated with different doses of IR.

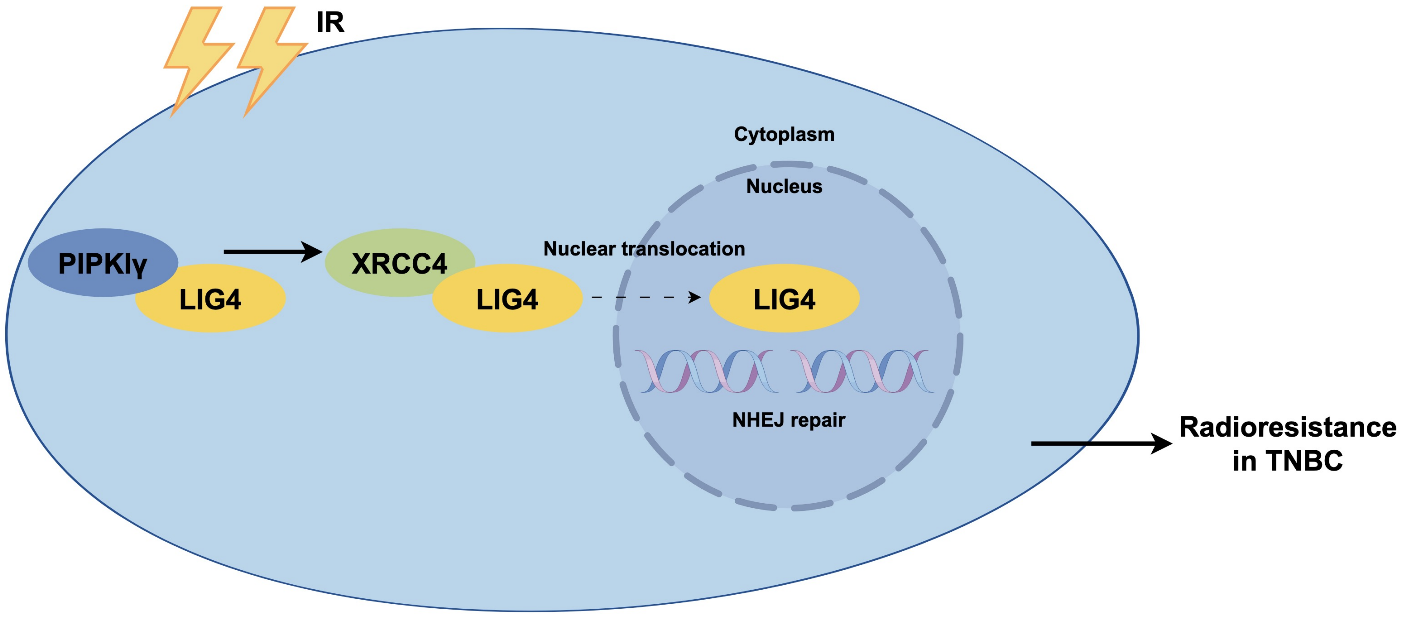

Supplementary Figure 5. Schematic illustration of the proposed mechanism by which PIPKIγ promotes NHEJ repair and contributes to radioresistance in TNBC. PIPKIγ interacts with LIG4 and enhances its association with XRCC4, thereby promoting the nuclear translocation of LIG4. This facilitates NHEJ-mediated repair of DSBs, ultimately contributing to enhanced radioresistance in TNBC. The figure was created with Figdraw (www.figdraw.com).
