# Supplementary material for: PIPKIγ promotes non-homologous end joining through LIG4 to enhance radiotherapy resistance in triple-negative breast cancer
Source: Cell Death Dis. 2025 Jul 31;16(1):578. doi: 10.1038/s41419-025-07894-5 (PMC12314031; doi:10.1038/s41419-025-07894-5)
Supplement: Supplementary file 2 — Supplementary Materials and Methods [file 41419_2025_7894_MOESM2_ESM.docx]

**Supplementary Materials and Methods**

**Reagents**

The antibodies used in this study included: anti-PIPKIγ (Cell Signaling, Cat. # 3296S), anti-Ku80 (ABclonal, Cat. # A5862), anti-Ku70 (ABclonal, Cat. # A0883), anti-LIG4 for WB (Proteintech, Cat. # 12695-1-AP), anti-LIG4 for IF (Abcam, Cat. # ab193353), anti-XLF (ABclonal, Cat. # A4985), anti-DNA-PKcs (Abcam, Cat. # ab70250), anti-Flag (ABclonal, Cat. # AE005), anti-GFP (Abcam, Cat. # ab290), anti-β-actin (Cell Signaling, Cat. # 4970), anti-Lamin A/C (ABclonal, Cat. # A19524), and anti-γH2AX (S139) (Cell Signaling, Cat# 9718).

For co-IP experiments, anti-DYKDDDK magnetic beads (Selleck, Cat. # B26102) and GFP-Trap (ChromoTek, Cat. # gta-100) were used. Cytoplasmic and nuclear proteins were extracted using the Nucleoprotein Extraction Kit (Solarbio, Cat. # R0050). SCR7 pyrazine (MCE, Cat. # HY-107845) was used to inactivate the enzyme activity of LIG4. UNC3230 (MCE, Cat. # HY-110150) was used to inactivate the canonical enzyme activity of PIPKIγ.

The sequences of the siRNAs used in this study are as follows:

si-PIPKIγ-1: 5'-AUCCGCGUCGUGGUCAUGAACAACA-3' (sense)

si-PIPKIγ-2: 5'-GCGUGGUCAAGAUGCACCUCAAGUU-3' (sense)

**Comet assay**

MDA-MB-231 cells, grown to 80–90% confluency, were collected and resuspended in PBS at a density of 2 × 10^5^ cells/ml. Cells were then processed using the alkaline comet assay, following the manufacturer’s protocol (Trevigen, Cat. # 4250050-K). The resulting data were analyzed with CometScore software (casplab_1.2.3), calculating the tail moment and the percentage of DNA in the tail to assess the degree of DNA damage.

**FACS analysis**

To determine NHEJ and HR efficiency, HCA2-I9a and HCA2-H15c cells underwent transfection with 5 μg of I-SceI vector, 15 ng of DsRed vector, along with plasmids or siRNAs. In a similar setup, alt-NHEJ efficiency was assessed in HCA2-hTERT cells using EJ2SceGFP reporter system. At 72h post-transfection or post-doxycycline induction, a minimum of 20,000 cells were collected for analysis on a BD FACSVerse flow cytometer (BD Biosciences, USA). The data were processed with FlowJo, with the ratios of GFP-positive and DsRed-positive cells serving as indicators of relative NHEJ or HR efficiency.

**Co-IP assay**

HEK293T cells, after transfection with the required plasmids, were harvested and lysed in a buffer containing 10 mM Tris/Cl (pH 7.5), 150 mM NaCl, 0.5 mM EDTA, 0.1 % SDS, 1 % Triton™ X-100, 1 % deoxycholate, and a protease inhibitor cocktail. After half an hour-incubation on ice, the lysates were sonicated and centrifuged at 13,000 rpm at 4 °C for 10 minutes. The supernatant was incubated with GFP-Trap or Flag-beads at 4 °C overnight. The beads underwent four washes with cold lysis buffer, and bound proteins were released by boiling the beads with 2× sample buffer for 10 minutes before Western blot analysis.

**GST pull-down assay**

Following induction with 0.1 mM Isopropyl β-D-Thiogalactopyranoside, GST-fusion and control proteins were produced in E. coli and subsequently isolated using glutathione Sepharose 4B beads (GE Healthcare, USA). These proteins were mixed with purified target proteins in a Tris-HCl buffer (10 mM, pH 8.0) supplemented with 1 mM EDTA and 100 mM NaCl, and incubated at 4 °C overnight. After incubation, the mixture was centrifuged and washed three times with cold buffer. The bound proteins were eluted by boiling the beads in 2× sample buffer for 10 minutes and analyzed via Western blot.

**Clonogenic assay**

Stable MDA-MB-231 and SUM159PT cells, either overexpressing or knocked out for PIPKIγ, were plated in six-well plates at 250 or 500 cells per well. After approximately two weeks of incubation, the cells were fixed with 4% paraformaldehyde and stained using 0.1% crystal violet. Colonies with a minimum of 50 cells were counted.

**EdU assay**

HCA2-I9a cells, either in a proliferating or confluent state, were analyzed using the EdU Cell Proliferation Kit with Alexa Fluor 555 (Epizyme, Cat. # CX003) following the manufacturer's protocol. Fluorescent images were acquired, and the percentage of EdU-positive cells was manually counted.

**Immunofluorescence assay**

Transfected cells were placed on coverslips and incubated in 12-well plates. After 24 or 48 hours, they were washed with cold PBS and fixed in 4% paraformaldehyde for 15 minutes. The cells were permeabilized with 0.25% Triton X-100 for 30 minutes and blocked with 2% bovine serum albumin for 1 hour. Primary antibody incubation occurred overnight at 4 °C, followed by secondary antibody incubation for 1 hour in the dark. After mounting with DAPI, images were captured using a Nikon confocal microscope.

Image analysis was performed using Image-Pro Plus software to quantify fluorescence intensities. Nuclear fluorescence intensity and total cellular fluorescence intensity were measured, and the relative nuclear intensity was calculated using the formula: Relative nuclear fluorescence intensity = Nuclear fluorescence intensity / Total fluorescence intensity.

**Generation of stable PIPKIγ overexpression and knockout cells**

HEK293T cells were transfected with plasmids, including pCDH-CMV and sgRNA vectors targeting PIPKIγ. After 72 hours, viral supernatants were collected and filtered through a 0.45 μm filter. The viral supernatants, supplemented with 6 μg/ml polybrene, were used to infect cells. Following a 48-hour infection period, cells were selected with antibiotics for 2 weeks: MDA-MB-231 and SUM159PT PIPKIγ overexpression cells with 10 μg/ml blasticidin, MDA-MB-231 and SUM159PT PIPKIγ knockout cells with 2 μg/ml puromycin, and CLZ3 PIPKIγ overexpression cells with 10 μg/ml blasticidin. Overexpression or knockout efficiency was confirmed by Western blot analysis. The sgRNA sequences targeting PIPKIγ are PIPKIγ-sg1: 5′-GCGCGCGGCCATGGAGCTGG-3′ and PIPKIγ-sg2: 5′-TGCAGGTTTGGCTCAGAAGA-3′.

**Xenograft assay**

Five-week-old female BALB/c nude mice were obtained from Zhejiang Vital River Laboratory (Zhejiang, China). Each mouse received a 100 μl subcutaneous injection of 1 × 10^6^ or 2 × 10^6^ MDA-MB-231 cells mixed with 20% Matrigel. Once tumors became palpable, mice were randomized into treatment groups (n = 6 per group) with comparable tumor volumes. Animals assigned to irradiation were anesthetized and immobilized. All body regions except the tumor were shielded with lead, and localized X-ray irradiation was delivered to the tumor site. Tumor sizes were monitored every three days. Tumor volume was calculated as length × width^2^ / 2. The relative tumor volume (RTV) was determined by the formula: RTV = (Tumor volume on measurement day) / (Tumor volume on day 0). The tumor growth inhibition rate was determined to assess the efficacy of treatment in reducing tumor progression. To assess tumor growth inhibition rate on day 18, the following equation was applied: tumor growth inhibition rate = [1 − (RTV of the treated group) / (RTV of the control group)] × 100. Sample sizes were chosen based on commonly used standards in the field and previous publications. No blinding was performed. Investigators were aware of group allocation during tumor measurement and analysis.

The study was conducted in accordance with the guidelines of the Laboratory Animal Care Committee at Tongji University, with the maximum tumor size limited to 15 mm.
