# Supplementary material for: PIPKIγ promotes non-homologous end joining through LIG4 to enhance radiotherapy resistance in triple-negative breast cancer
Source: Cell Death Dis. 2025 Jul 31;16(1):578. doi: 10.1038/s41419-025-07894-5 (PMC12314031; doi:10.1038/s41419-025-07894-5)

Figure 1D

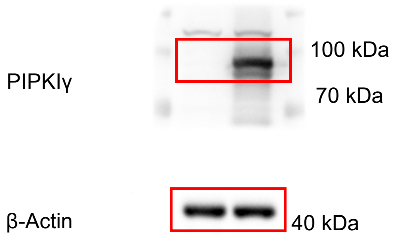

Figure 1E

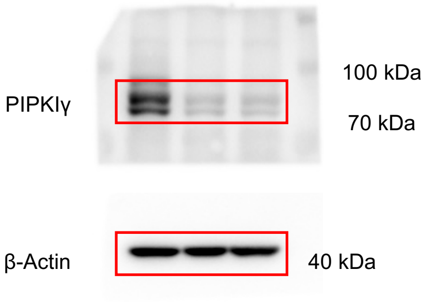

Figure 1G

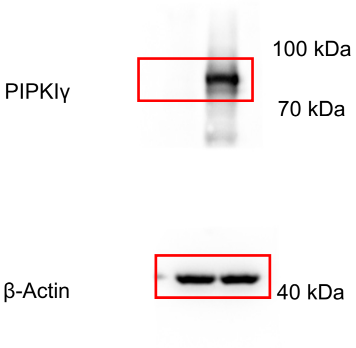

Figure 1H

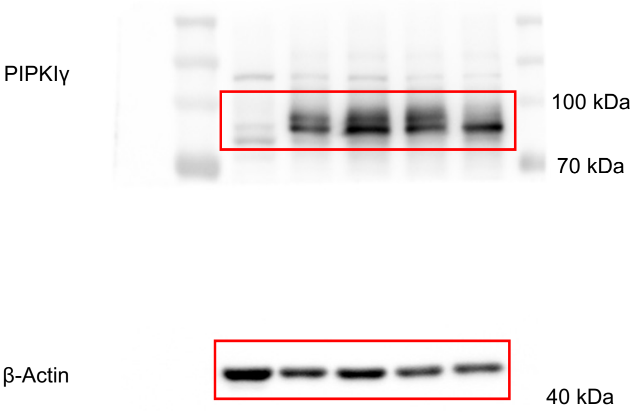

Figure 1I

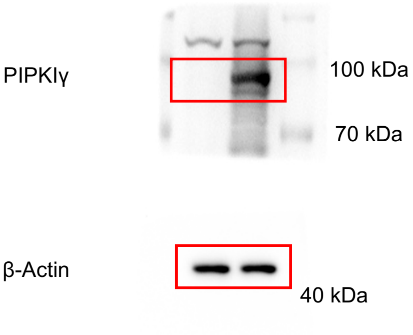

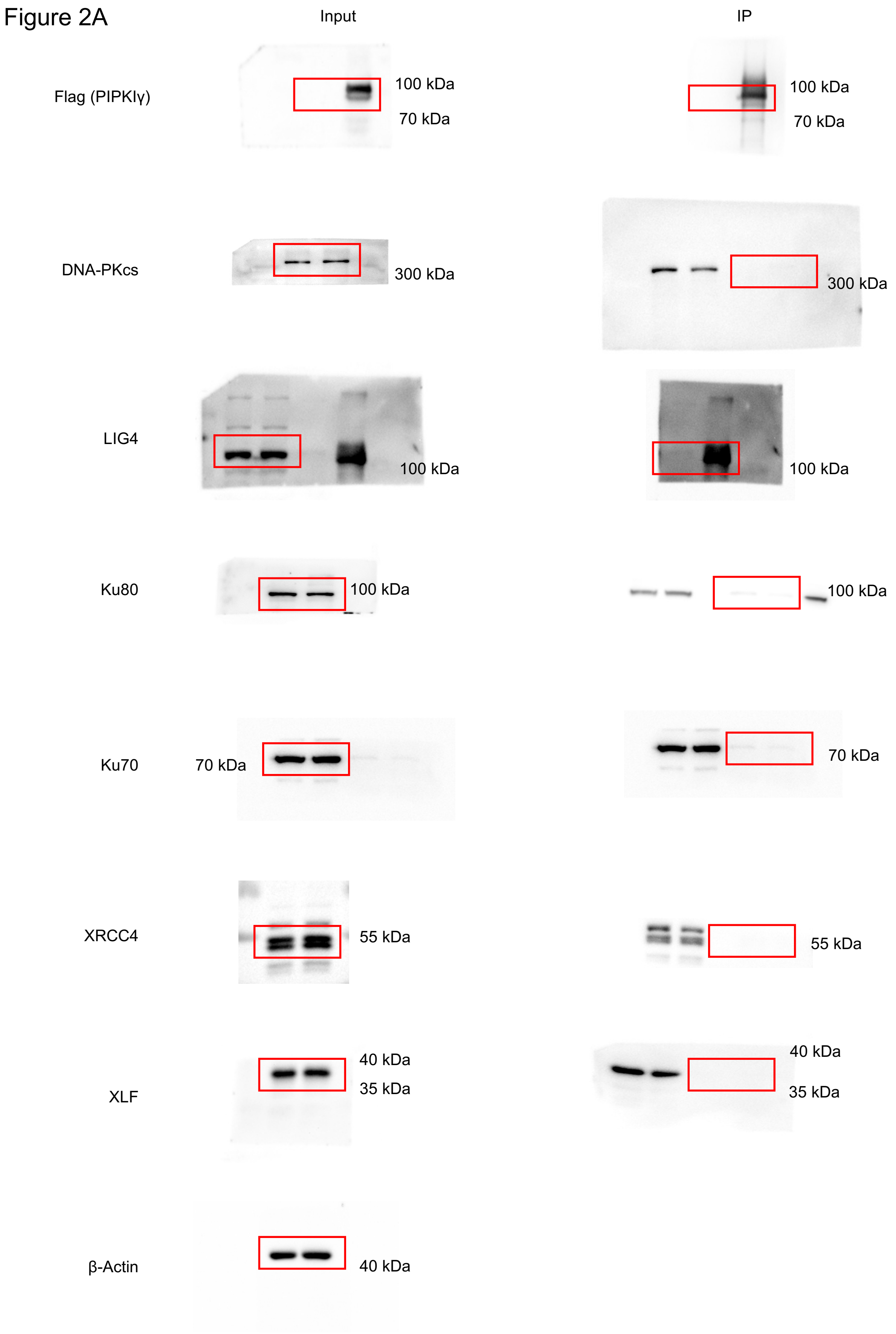
Figure 2A
Input
IP
Flag (PIPKIγ)
100 kDa
70 kDa
DNA-PKcs
300 kDa
LIG4
100 kDa
Ku80
100 kDa
Ku70
70 kDa
XRCC4
55 kDa
XLF
40 kDa
35 kDa
β-Actin
40 kDa

Figure 2B

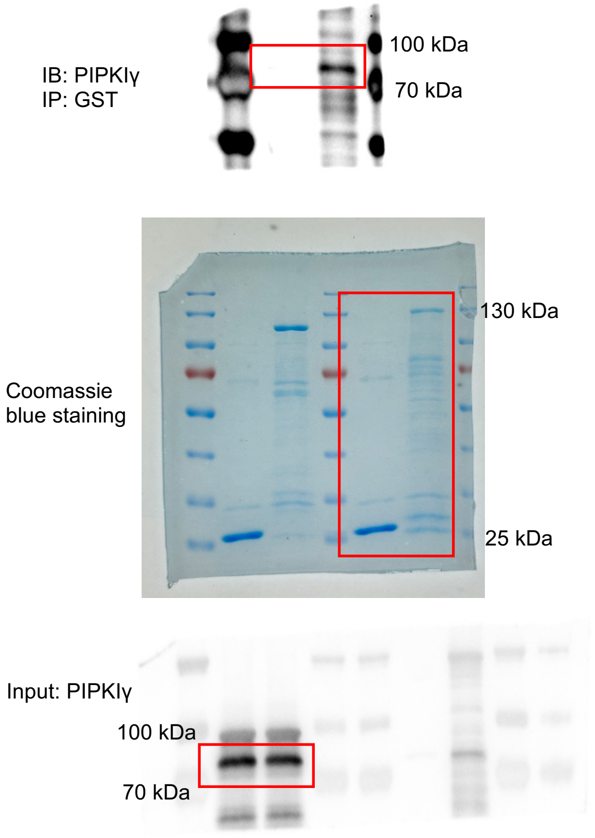

Figure 2E

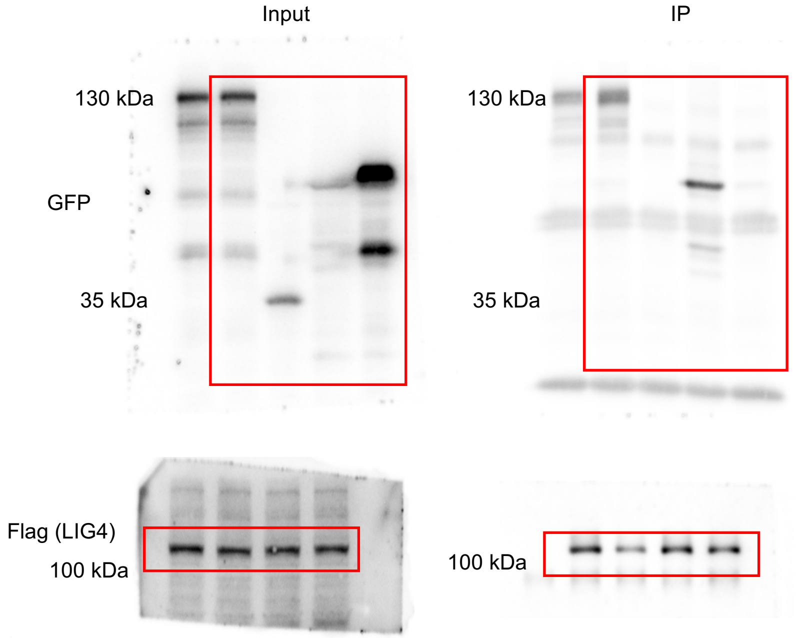

Figure 2F

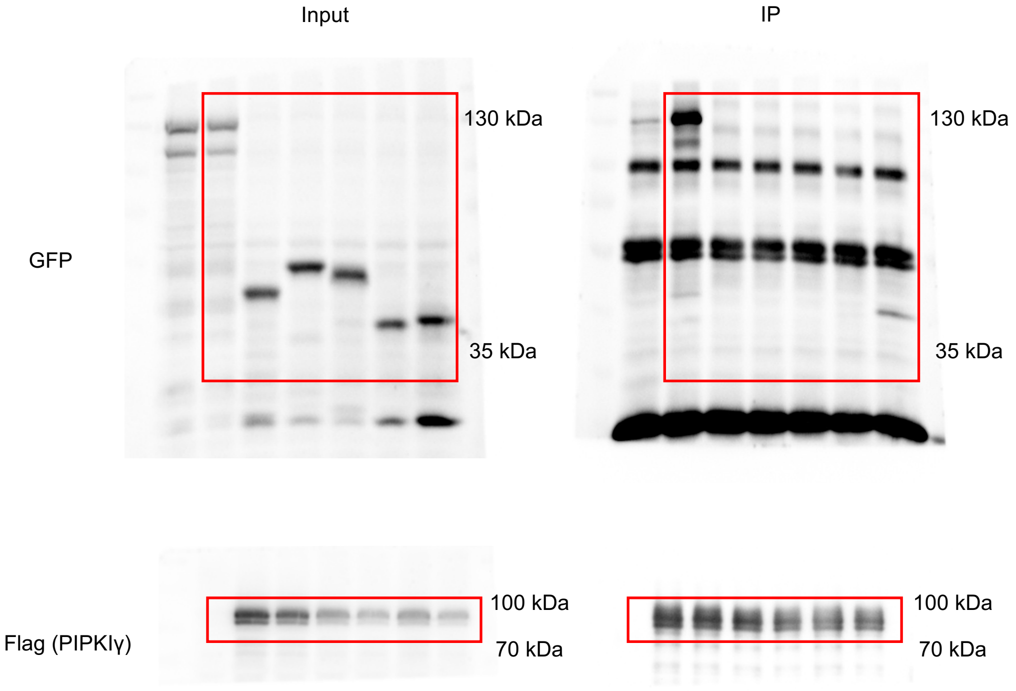

Figure 2G

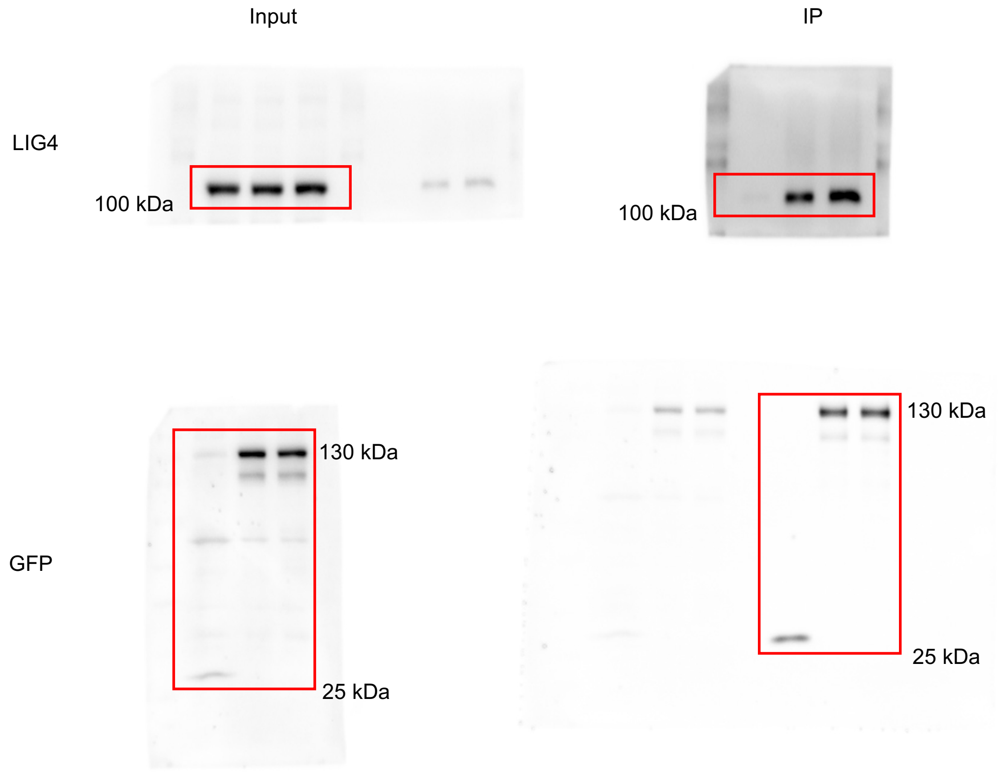

Figure 2H

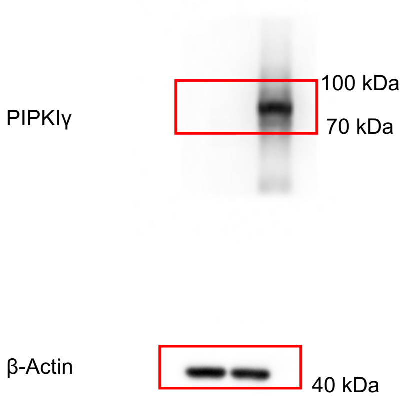

Figure 3A

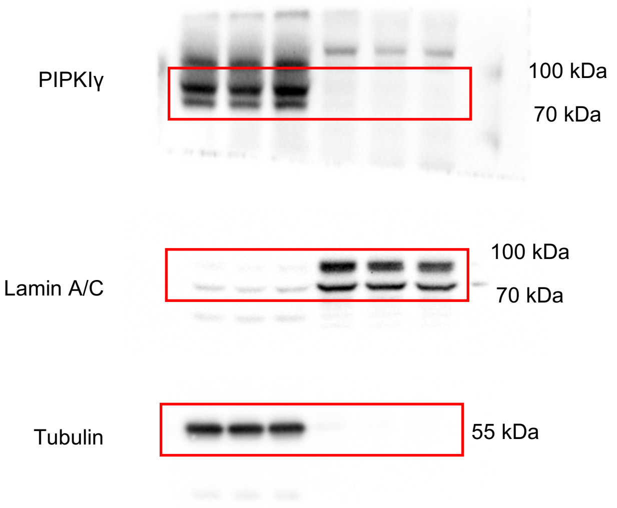

Figure 3B

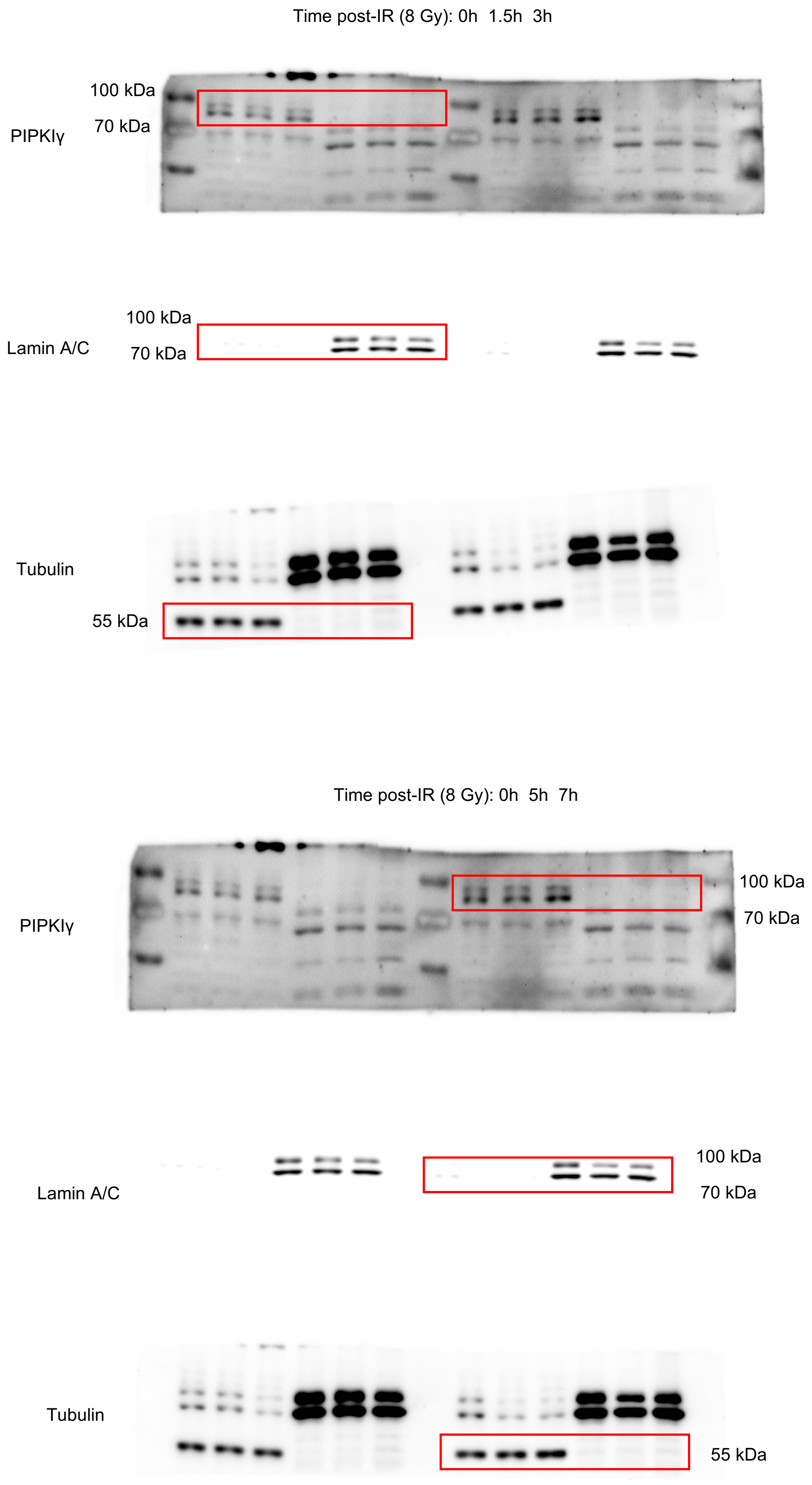
Time post-IR (8 Gy): 0h 1.5h 3h
100 kDa
70 kDa
PIPKIγ
100 kDa
Lamin A/C
70 kDa
Tubulin
55 kDa
Time post-IR (8 Gy): 0h 5h 7h
100 kDa
70 kDa
PIPKIγ
100 kDa
Lamin A/C
70 kDa
Tubulin
55 kDa

Figure 3C

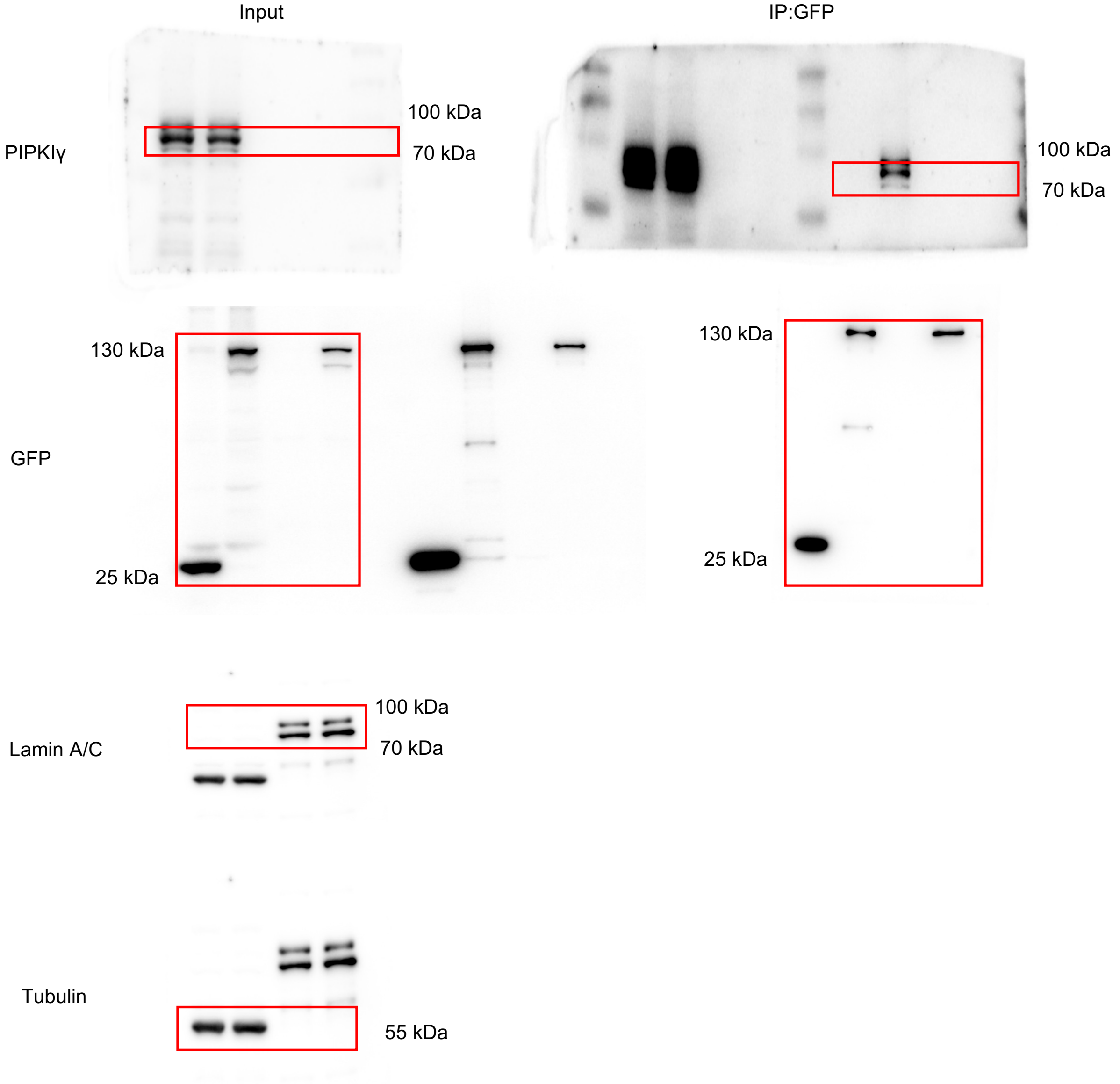

Figure 3D

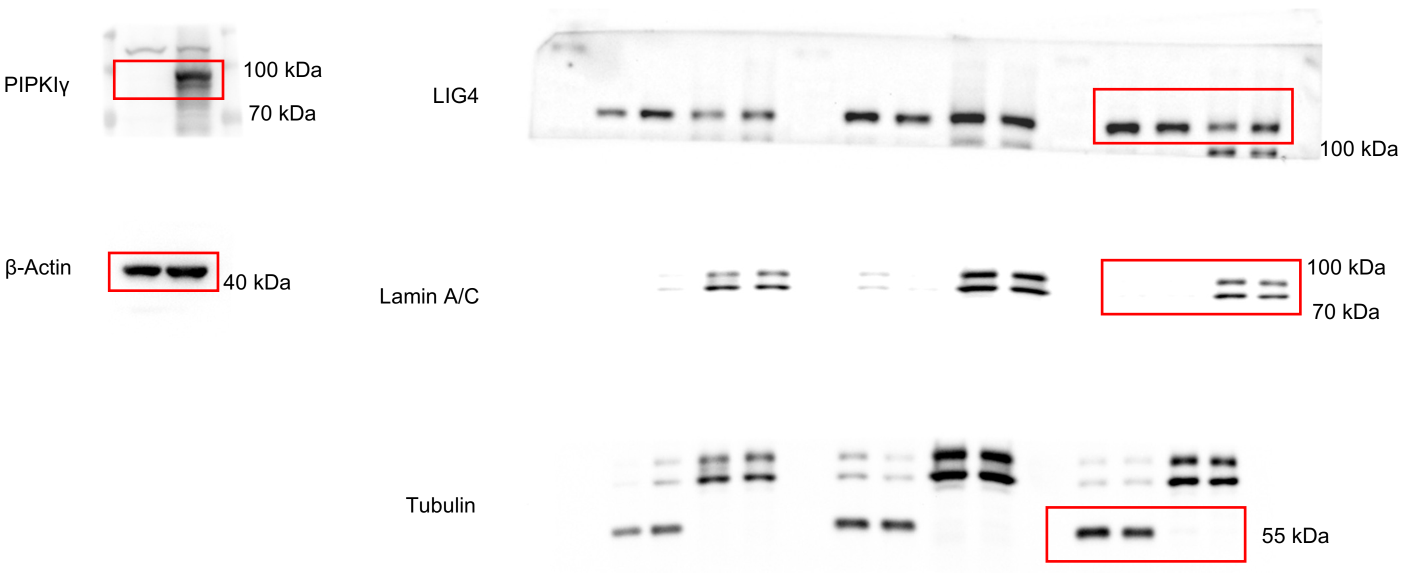

Figure 3E

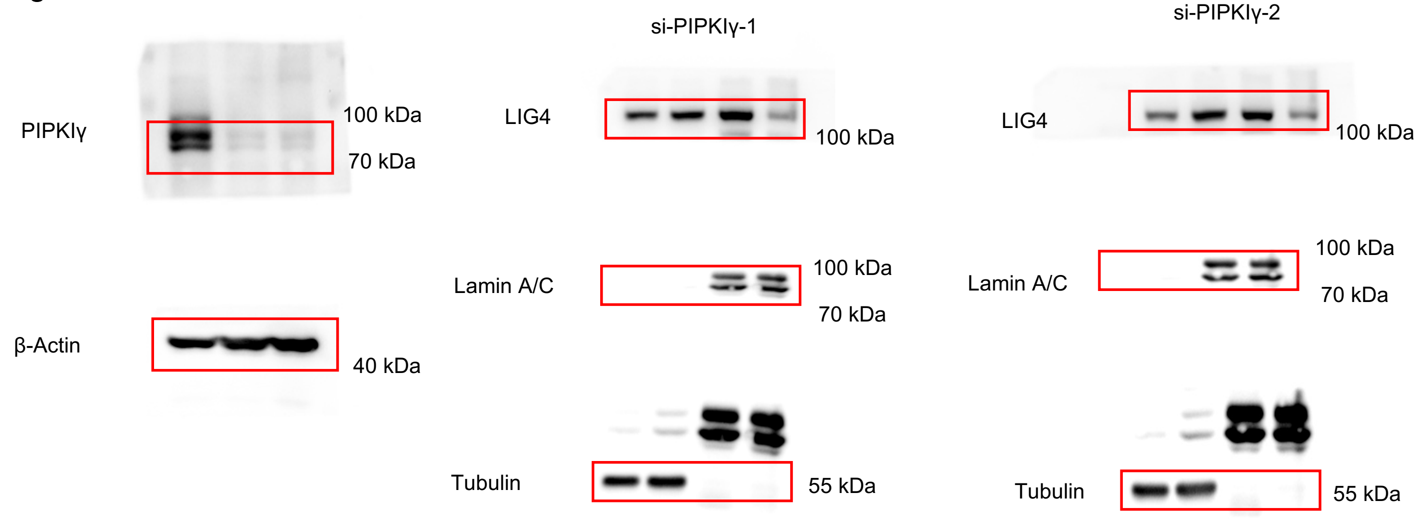

Figure 3F

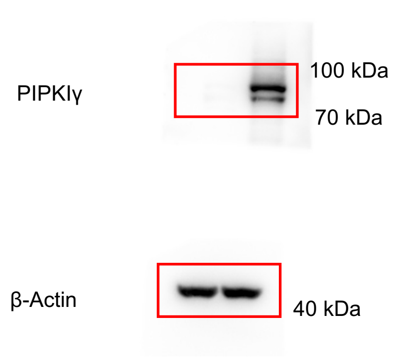

Figure 3G

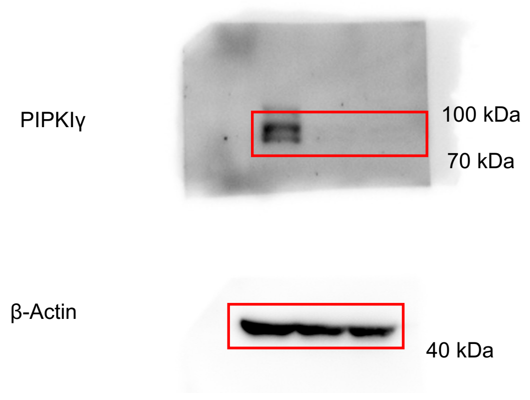

Figure 3H

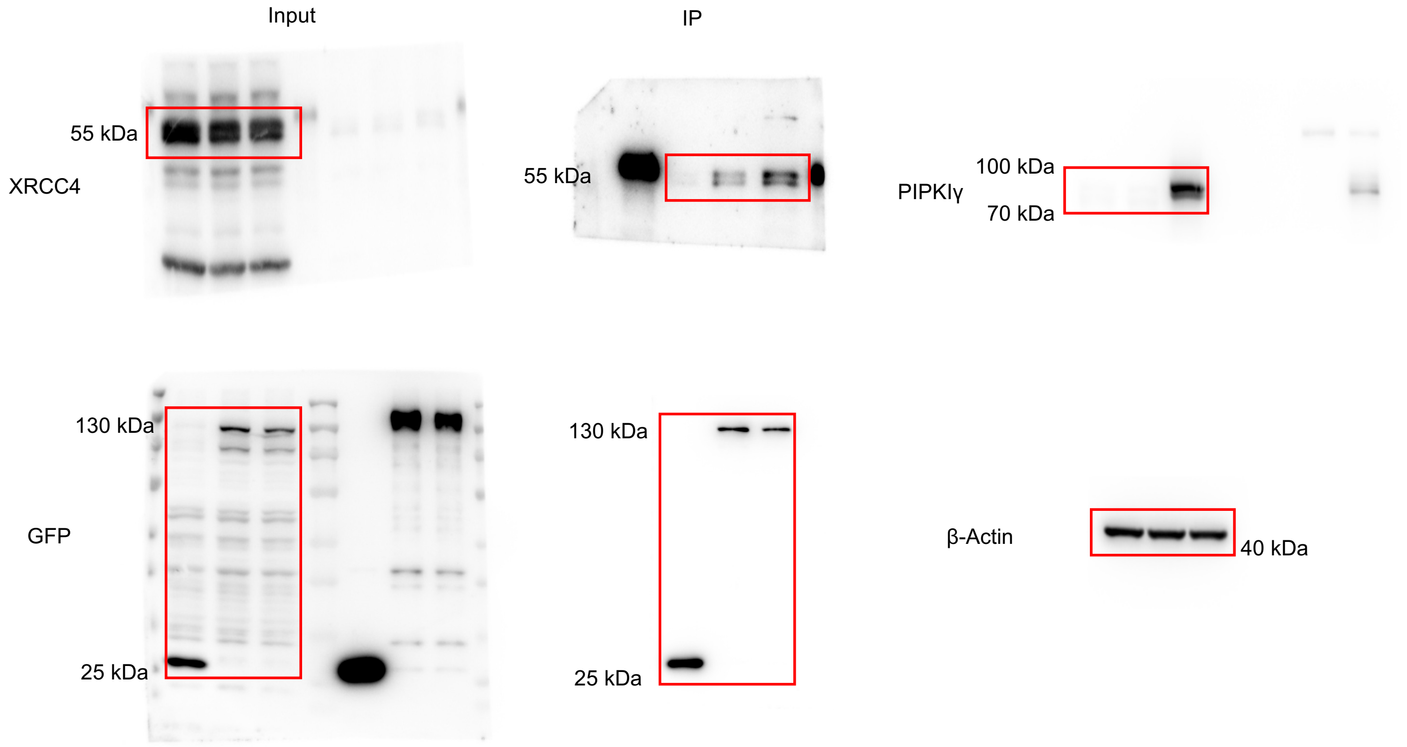

Figure 3I

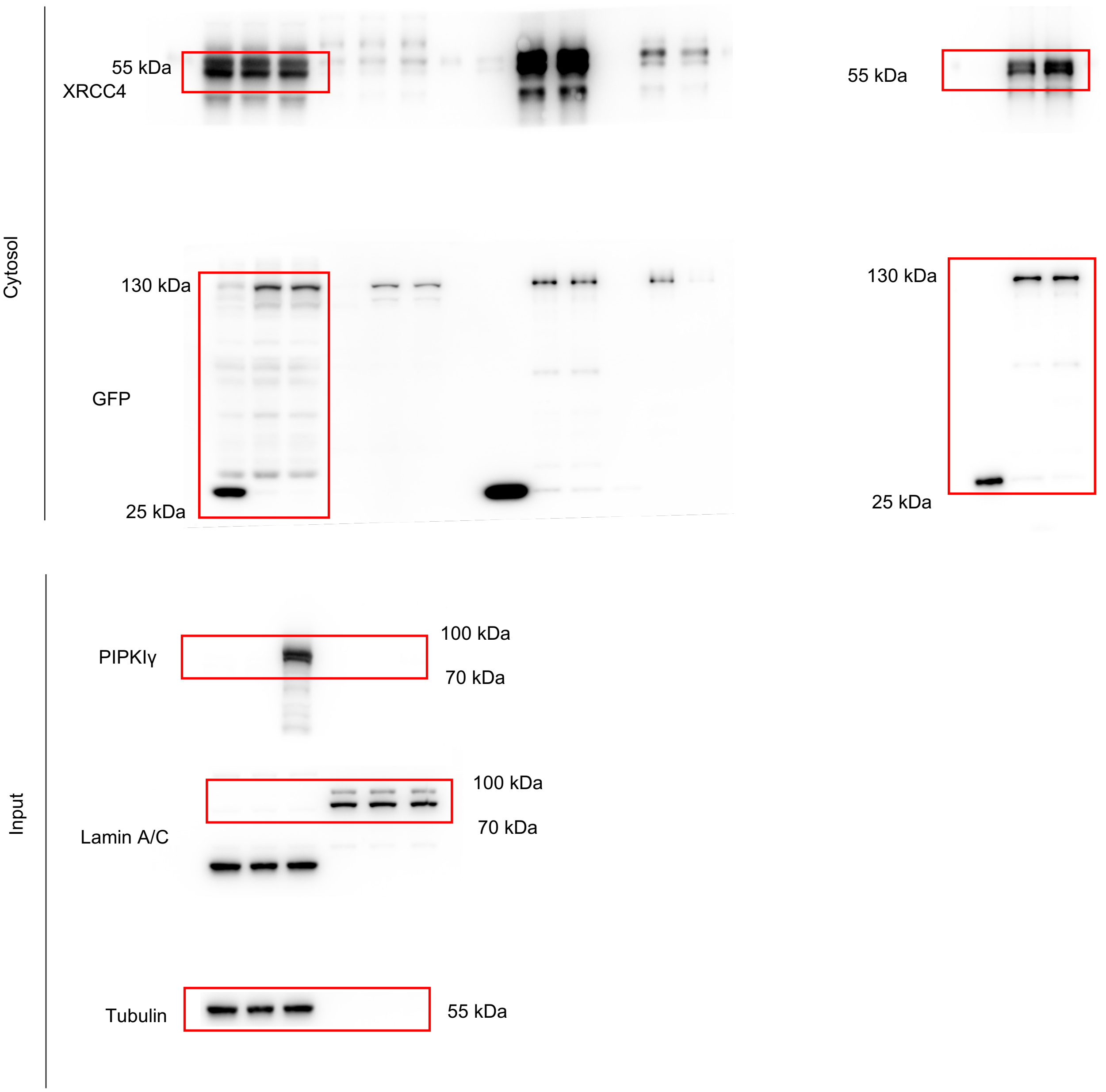

Input
IP:GFP
55 kDa
XRCC4
55 kDa
130 kDa
GFP
25 kDa
130 kDa
25 kDa
Cytosol
PIPKIγ
100 kDa
70 kDa
Lamin A/C
100 kDa
70 kDa
Tubulin
55 kDa
Input

Figure 3J

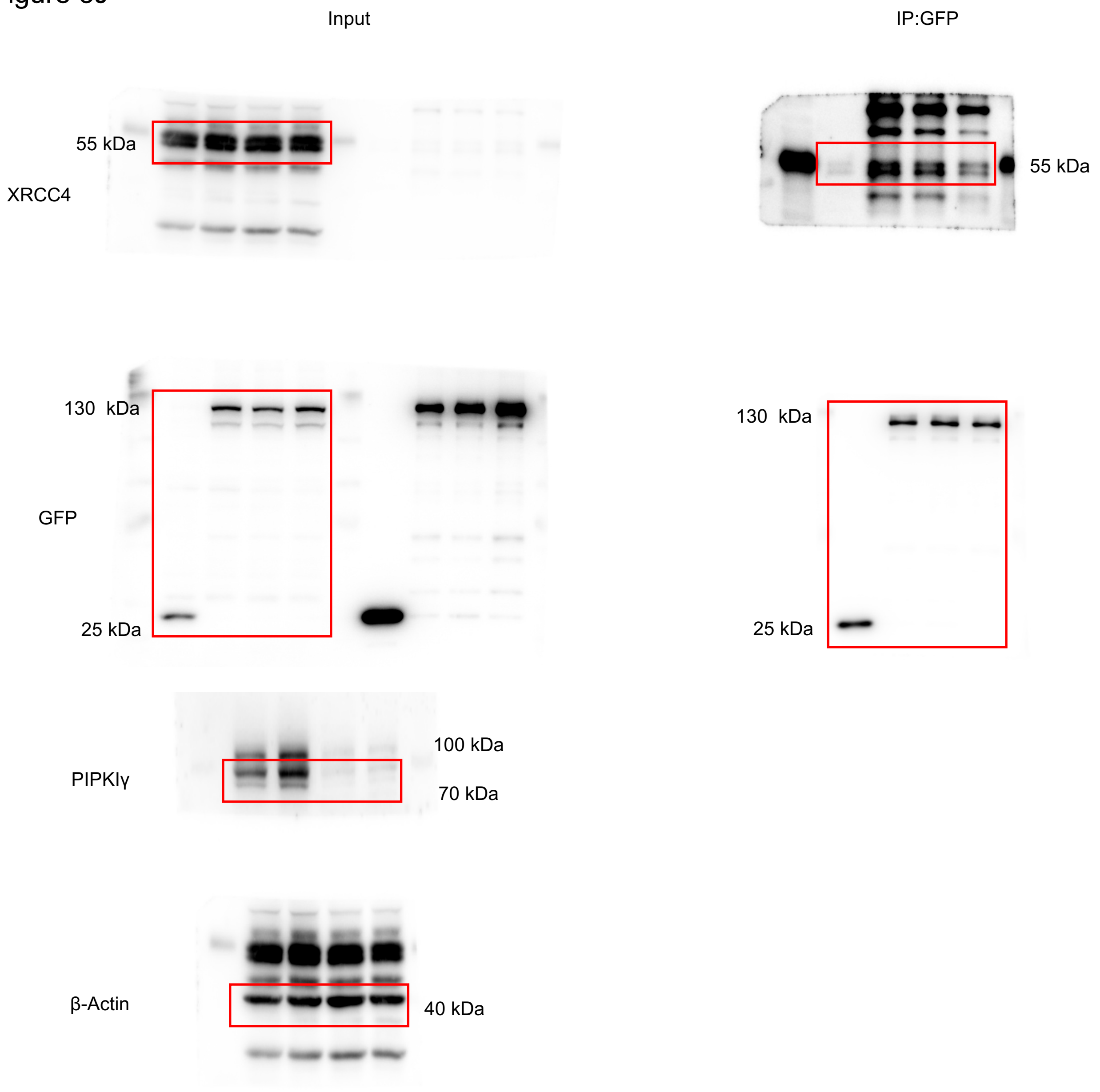

Figure 4A

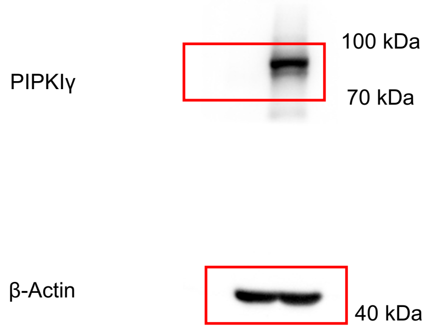

Figure 4B

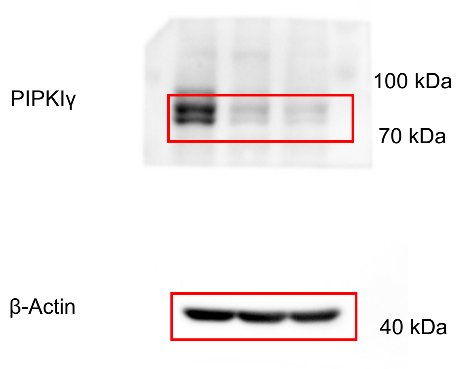

Supplementary figure 1D

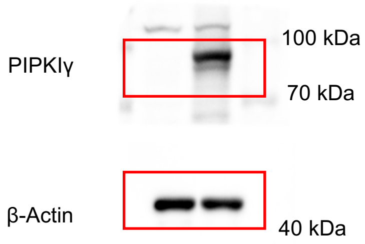

Supplementary figure 2B

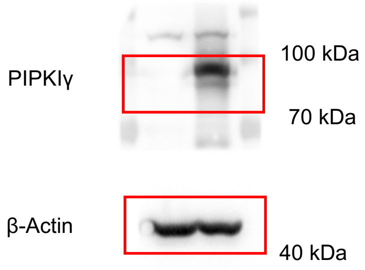

Supplementary figure 2D

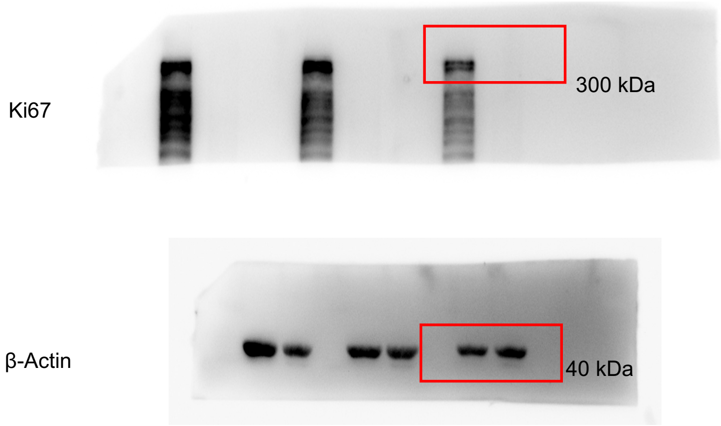

Supplementary figure 3B

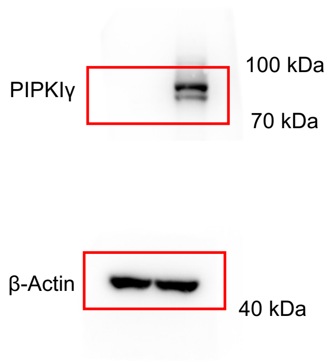

Supplementary figure 3C

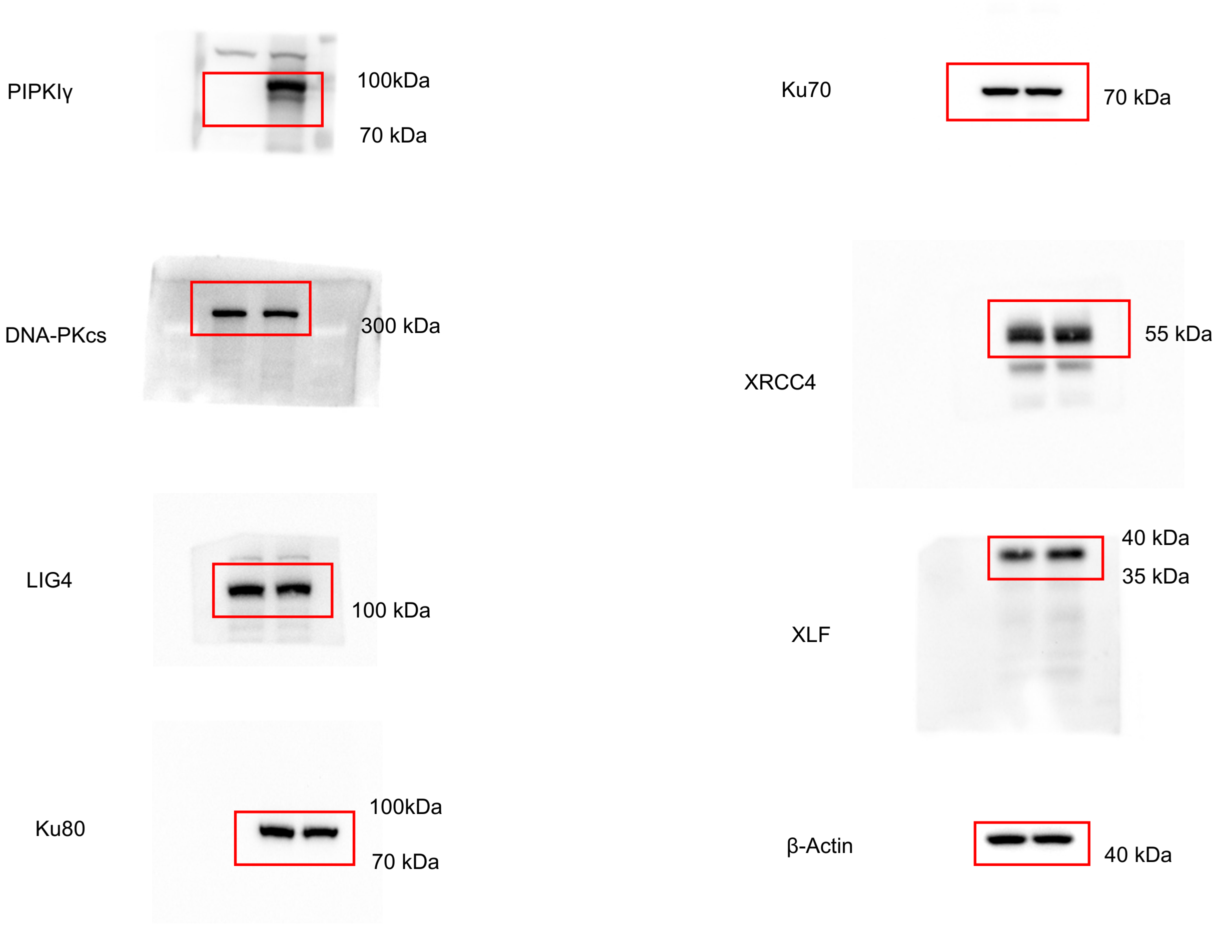

Supplementary figure 3D

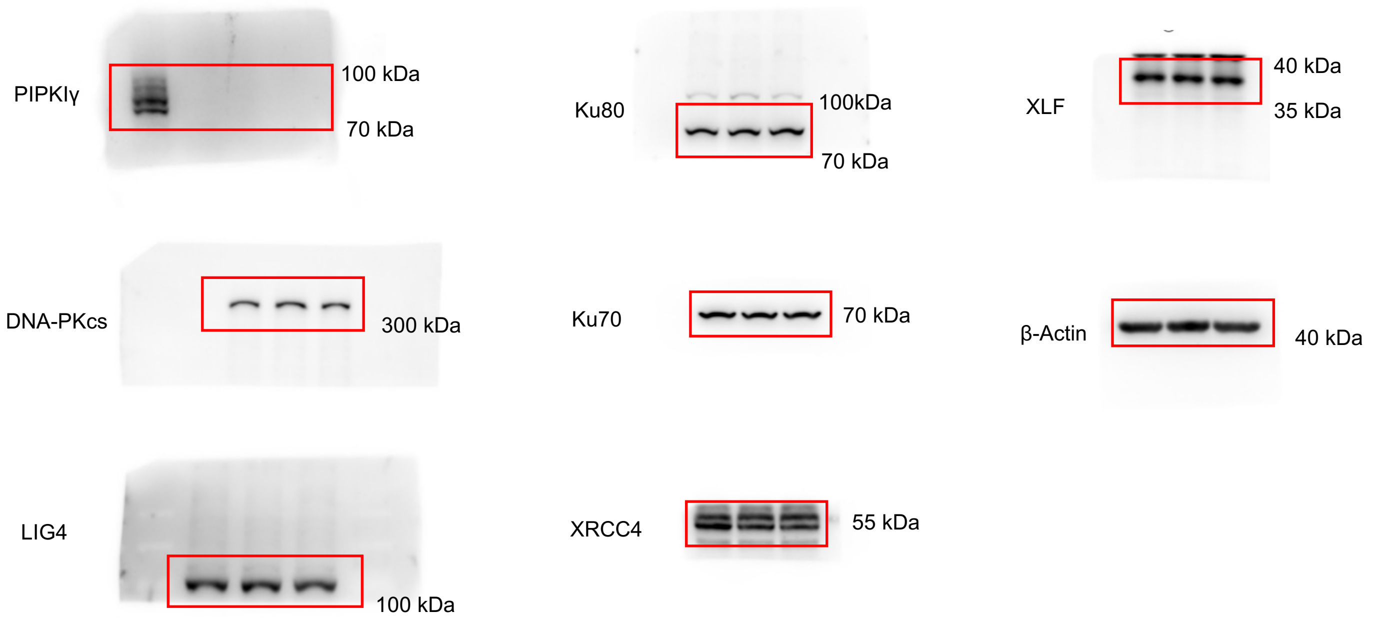

Supplementary figure 4A

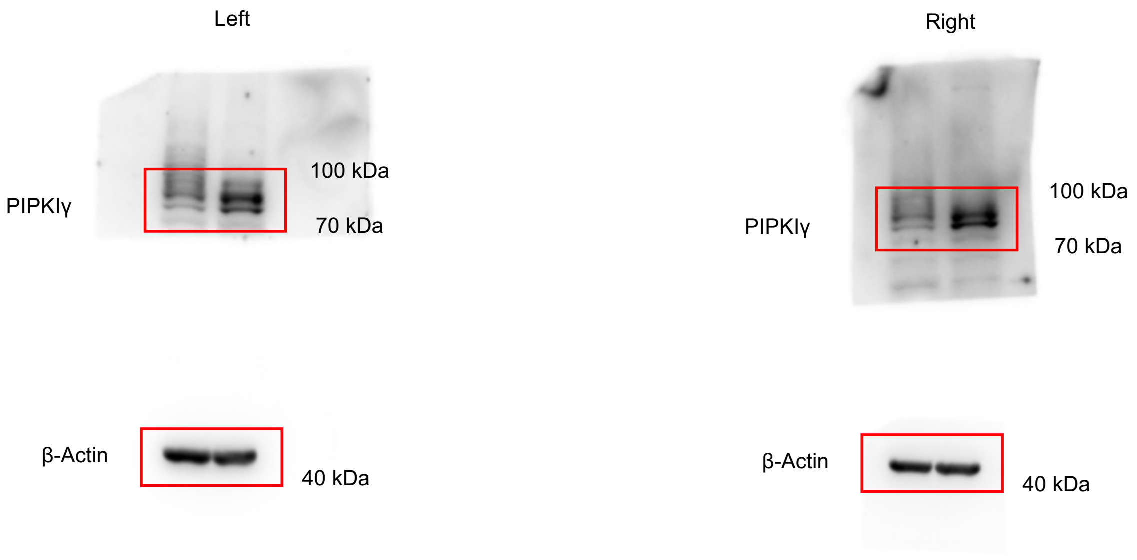

Supplementary figure 4F

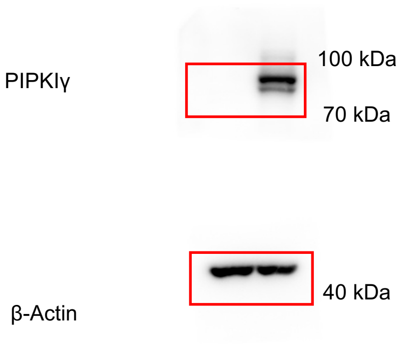

Supplementary figure 4G

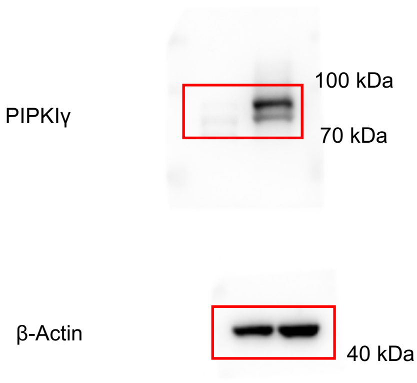

Supplementary figure 4H

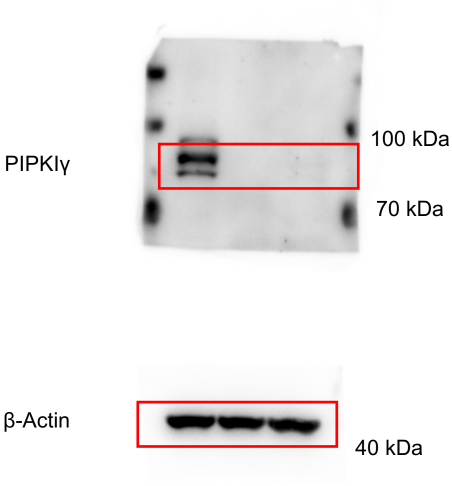

Supplementary figure 4I

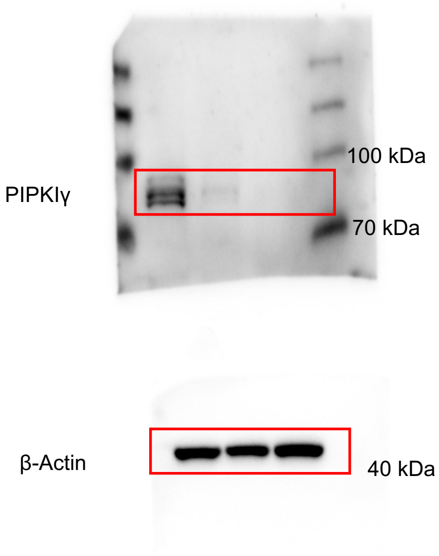

Supplement: Supplementary file 3 — Original Western Blots [file 41419_2025_7894_MOESM3_ESM.pdf]
